# Integration of visual context in early and late bilingual language processing: evidence from eye-tracking

**DOI:** 10.3389/fpsyg.2023.1113688

**Published:** 2023-04-26

**Authors:** Dato Abashidze, Angela Schmidt, Pavel Trofimovich, Julien Mercier

**Affiliations:** ^1^Leibniz-Centre for General Linguistics, Berlin, Germany; ^2^Education Department, Concordia University, Montreal, QC, Canada; ^3^Département d' éducation et formation spécialisées, Université du Québec à Montréal, Montreal, QC, Canada

**Keywords:** bilingualism, recent-event preference, eye-tracking, spoken sentence comprehension, executive function

## Abstract

Previous research on the processing of language embedded in a rich visual context has revealed the strong effect that a recently viewed action event has on language comprehension. It has been shown that listeners are more likely to view the target object of a recently performed event than look at the target object of a plausible future event during sentence utterance, regardless of the tense cue. In the current visual-world eye-tracking experiments, we tested the strength of the recently observed visual context with a group of English monolingual and two groups of English–French early and late bilingual speakers. By comparing these different groups, we examined whether bilingual speakers, as a consequence of greater cognitive flexibility when integrating visual context and language information, show early anticipatory eye-movements toward the target object. We further asked whether early and late bilinguals show differences in their processing. The findings of the three eye-tracking experiments revealed an overall preference for the recently seen event. However, as a result of the early provision of tense cue, this preference was quickly diminished in all three groups. Moreover, the bilingual groups showed an earlier decrease in reliance on the recently seen event compared to monolingual speakers and the early bilinguals showed anticipatory eye-movements toward the plausible future event target. Furthermore, a post-experimental memory test revealed that the bilingual groups recalled the future events marginally better than the recent events, whereas the reverse was found in the monolingual groups.

## 1. Introduction

Much of current psycholinguistic research has shown that the context in which language occurs influences the interpretation and production of language as it unfolds. The rapid integration of visual stimuli during the processing of spoken utterances has been the subject of numerous investigations. Findings have shown anticipatory eye-movements toward a target object before it was explicitly mentioned as a result of the presentation of linguistic or visual factors, which influenced the interpretation of utterances (e.g., Tanenhaus et al., 1995; Kamide et al., 2003; Chambers et al., 2004; Otten et al., 2007). Studies investigating the effect of visual context on prediction when comprehenders were provided with linguistic and visual input have revealed the impact, at the speed of milliseconds, that viewing has on the processing of language (e.g., DeLong et al., 2005; Altmann and Kamide, 2007; Knoeferle et al., 2011). Language processing would therefore seem to be finely attuned to the interplay of any visual and linguistic factors within a given context (see Rigoli and Spivey, 2015, for an overview). When we consider language processing in bilingual speakers, these factors gain new perspectives, as findings from numerous studies have revealed that the multiple languages of a bilingual are always activated (e.g., Spivey and Marian, 1999; Weber and Cutler, 2004). Furthermore, research has shown processing effects related to the age of language acquisition (e.g., Wartenburger et al., 2003; Saur et al., 2009) and the degree of language proficiency (e.g., Blumenfeld and Marian, 2007). Bilinguals have also demonstrated an enhanced performance during complex visual search tasks (e.g., Friesen et al., 2015; Hartsuiker, 2015). It is therefore probable that bilingual and monolingual speakers differ in how language information within a rich visual context is processed.

### 1.1. Visual context and monolingual language processing

When considering the impact of visually displayed action events on language comprehension, recent eye-tracking studies of German have revealed that participants rely more on a shown recent event rather than anticipate a plausible future event when hearing related utterances. In an experimental setting, participants viewed a performed action (e.g., “sweetening pancakes”) and then listened to a sentence (NP1-Verb-Adv-NP2) that described this action as having been performed in the recent past or that it was to take place in the immediate future. A bias, termed the recent-event preference, was revealed, in that participants inspected the depicted target of the recently performed event more often than the other plausible target object (that might be involved in a future action) shown in the display irrespective of past or futuric present tense cue (e.g., Knoeferle and Crocker, 2007; Abashidze et al., 2011). These experiments were conducted in German, and sentences were constructed with a verb in either the past or the futuric present tense followed by a temporal adverb in both the past condition (e.g., *zuckerte gerade*, “sweetened recently”) and the futuric present condition (e.g., *zuckert gleich*, “sugars soon”). Interestingly, an overall significant decrease in the reliance on the recent event could not be achieved by manipulations of the frequency and actor's gaze cue. In the follow-up studies, for instance, Abashidze et al. (2019) adjusted the frequency of past vs. future conditions in favor of the future event (the future condition was shown in 75% of the trials). In another study, actor's gaze cue was pitted against the recent event preference and was used to guide participants' looks toward the future target object (i.e., Abashidze and Knoeferle, 2021). While these later manipulations affected participants' eye-movements toward the recent-event target, neither resulted in the future event being overall favored by participants.

Eye-tracking experiments investigating the integration of concurrent visual and linguistic stimuli have shown the effect that real-world and event knowledge have on eye-gaze patterns. For example, Chambers and San Juan (2008) found that perceptual information may be subordinate to communicative factors and event knowledge. These authors also showed that in a task where participants were required to combine various objects in a display, they were less likely to re-fixate on objects that they had used to form part of what might be considered a coherent whole. The work of Altmann and Kamide (2007) revealed the effect that tense cue has on comprehenders' real-world knowledge of a target object's affordances, in other words, the actions and properties connected with an entity, during language processing. They showed that anticipatory looks to objects in the visual display were guided by utterances denoting whether an action had already taken place or not. The authors argue that these looks show no bias for looking at a future action (what will happen next). Rather, they reveal the strong influence that the interplay between the information encapsulated by both verb tense and viewed object has on anticipatory eye-movements. The work of Knoeferle and Crocker (2007), however, found that the effect of tense cue was relatively weak. They postulated that visually depicted events, particularly when they are present throughout sentence utterance, strongly inform sentence interpretation. Furthermore, in previous findings, Knoeferle and Crocker (2006) showed that language comprehenders preferred to ground verb information through verification against a visual scene. They also showed the strength of visual information over real-world knowledge (albeit stereotypical) in sentence processing. As regards the establishment of verb-grounding, various authors found further evidence of this, confirming that being able to anchor a heard action in a visual scene takes precedence over the anticipation of what may come next (e.g., Abashidze et al., 2011, 2019; Knoeferle et al., 2011; Abashidze and Knoeferle, 2021), even though one might suppose that an action that has already been performed in the past is unlikely to happen again.

When considering the strength of visual and linguistic cues in language processing, one might consider, as do, among others, Saryazdi et al. (2018), the effect of the type of visual stimuli used in experimental settings. In many of the studies that revealed a recent-event preference, real-world actions (e.g., Abashidze et al., 2011; Knoeferle et al., 2011) and/or short video clips were used (e.g., Abashidze et al., 2011, 2019; Abashidze and Knoeferle, 2021). The rich visual context cues of real-world actions might have influenced comprehenders' eye-gaze patterns in a manner that differs from visual displays using more static images. Another factor that might explain the recent-event preference found in the above studies was that the linguistic stimuli was presented in German and the clear disambiguation of the sentences toward a future or past event took place relatively late in the sentence (during the temporal adverb). To observe whether the recent-event preference found with speakers of German in their native language could be replicated in another language, Abashidze and Chambers (2016) conducted a pilot study using the same visual material but with English as the linguistic stimuli (utilizing both statement and question sentences). Findings from this pilot study revealed that gaze toward the target image occurred earlier compared to the German studies. Furthermore, this eye-movement pattern occurred earlier in the question sentences than in the statement sentences, as the future or past tense (e.g., will/has sugar/ed) was introduced through an auxiliary verb preceding the main verb.

While the above studies show that recently observed event influence language processing, and monolingual comprehenders rely on the visual context heavily (e.g., Knoeferle et al., 2011; Abashidze and Chambers, 2016; Abashidze et al., 2019), less is known regarding bilingual language processing embedded in such rich visual context. The following sections, therefore, examine findings from research with a focus on the language experience particular to bilinguals speakers—such as simultaneous multiple language activation, language proficiencies, age of onset of acquisition, and advantages or disadvantages in executive functioning—that provide insights into reasons why bilingual speakers might process rich visual cues in the presence of language information differently from their monolingual counterparts.

### 1.2. Bilingual language experience and executive functioning

Findings from numerous eye-tracking studies involving bilingual speakers show that bilinguals activate both languages when phonological overlapping occurs between both languages (e.g., Spivey and Marian, 1999; Ju and Luce, 2004; Weber and Cutler, 2004). The same holds true for orthographic similarities, as shown in the study by Mishra and Singh (2014) on English-Hindi bilinguals during eye-tracked reading tasks.^1^ When Dutch–English–French trilinguals read cognates/noncognates, they showed a facilitation effect for cognates when comprehending in the second language and even in the third language for very proficient participants (Van Hell and Dijkstra, 2002). This cognate facilitation effect across three languages for trilinguals was also found in the work of Lemhöfer et al. (2004), irrespective of the language context.

Visual context is a further factor that plays a role in the activation and processing of a bilingual's languages. Hartsuiker (2015) reviewed research on whether visual information affects the degree of activation of a bilingual's languages. The results showed that a specific language becomes more or less activated depending on the visually presented contextual cues. Research by Chabal and Marian (2015) revealed between-language competition during the inspection of visual displays even when no linguistic input was provided. When examining language processing across modalities, Shook and Marian (2012) found that also bimodal bilinguals exhibit signs of competition between their languages, suggesting that both bottom-up and top-down connections can influence competition and dual language activation. Evidence for both top-down (setting) and bottom-up (language characteristics) processing was also found in the work of Shabani-Jadidi on multilingual perception (Shabani-Jadidi, 2016).

As the above studies illustrate, the languages of bilinguals appear to be simultaneously activated in any given context. As a result, speakers who move within various languages may need to monitor incoming linguistic and visual information in ways that differ from monolinguals. It has been speculated that this experience is the underlying cause for the enhanced executive function skills found with bilinguals (e.g., Green, 1998), as research has shown that speakers of more than one language are advantaged in their ability to perform tasks that require cognitive flexibility in both linguistic (e.g., Blumenfeld and Marian, 2011; Ikizer and Raḿırez-Esparza, 2018) and non-linguistic contexts (see Bialystok, 2018, for a review). Furthermore, they appear to be better at reducing their reliance on unimportant information, finding an alternative solution to an issue (e.g., Bialystok and Martin, 2004; Vega-Mendoza et al., 2015, for children and adults, respectively), and acquiring a new language faster (e.g., Bartolotti and Marian, 2012).

Some research into the processing of visual stimuli has shown differences between monolingual and bilingual speaker groups. Marian (2009) examined evidence from various studies and pointed out that bilinguals may be more sensitive to cross-modal factors than monolinguals during the processing of linguistic information. A study by Friesen et al. (2015) examined the reaction time and accuracy of bilinguals and monolinguals engaged in a visual search task. Findings from this study indicated that bilinguals performed better in trials of higher complexity in which target images differed from the distractor items in two features, shape and color (vs. one, in the less complex condition). While exploring the effect that visual and linguistic stimuli have on participants fixation of a target image in a visual world paradigm, Chabal et al. (2015) found that bilinguals outperformed their monolingual counterparts in more quickly locating the correct target. However, when Ratiu et al. (2017) used eye-tracking and reaction time measures to determine if there was a bilingual advantage in those aspects of executive functioning that guide visual attention, decision making, and goal maintenance, no advantage was found.

When comparing executive function and working memory performance between highly matched monolingual and simultaneous bilinguals (from a naturalistic bilingual setting), Antón et al. (2019) found that bilinguals outperformed monolinguals in some of the tasks that tested working memory but not in those that tested executive functioning. Kazemeini and Fadardi (2016), however, found evidence of an advantage for Kurdish–Persian early bilingual young adults in both memory test (Backward Digit Span Test) and executive control (Stroop Color Word task) tasks. Findings from a study by Wodniecka et al. (2010), examining the effects of bilingualism on memory performance, revealed that younger adults outperformed older adults, but that there was very little evidence for a bilingual advantage between the younger groups. When Yang and Yang (2017) tested Korean–English bilinguals and English native speakers with tasks meant to determine thier controlled processing ability (which affects both working memory and executive functioning), bilinguals showed an advantage.

Some studies show a disadvantage for bilinguals in performing tasks (in particular production tasks) that rely on verbal memory, due to lessened lexical resources, as a result of a smaller vocabulary and a diminished readiness of the lexical access (Gollan et al., 2005; Bialystok, 2009). However, when Kousaie et al. (2014) investigated an equally proficient French–English bilingual group, they found no advantage in executive functioning tasks but also no disadvantage on language tasks. Similarly, Kerrigan et al. (2017) found no group differences between bilinguals and monolinguals in verbal tasks. These authors additionally tested both groups in a Corsi block task, a task meant to assess working memory, and they performed similarly. The authors did, however, find that bilinguals outperformed monolinguals in a task that assessed the speed and accuracy of noticing visual changes in a display. Kerrigan et al. (2017) and Rosselli et al. (2019) describe an observed advantage for bilinguals in tasks that rely on visuo-spatial memory; under the stipulation that these results may be highly task- and group-dependent (in this regard, see also Luo et al., 2013).

The diversity of results in the above studies emphasizes the need to carefully assess multiple factors—such as the language groups and experimental tasks used (for example, as Dijkstra et al., 1998, indicate that even the level of bilingual activation is task-dependent)—when interpreting findings from bilingual language processing. Nonetheless, these studies indeed show that, in many experimental settings, bilinguals function differently than monolinguals. Due to the co-activation of a bilinguals' various languages, a constant inhibition of other-language competitor information is required. Furthermore, bilingual speakers must constantly monitor and update the contexts in which their languages are used. This may lead to enhancing the cognitive abilities that are used in executive functions in a way that differs from monolinguals. Navigating multiple languages and contexts simultaneously might furthermore result in a working memory advantage. The bilingual experience might additionally be modified by both the length of time and degree of use of a bilingual's languages. As Blumenfeld and Marian (2007) point out, these factors may alter the degree of parallel activation during multi-language processing and consequently impact the executive function of bilinguals.

### 1.3. Early vs. late bilinguals: Effects of age of language acquisition and proficiency on language processing

The possible correlation between ultimate achievement of a second language and the age at which one begins acquiring a language has been the subject of numerous studies. Earlier research posited puberty as the time frame after which native-like achievement becomes more difficult (e.g., Johnson and Newport, 1989; DeKeyser, 2000). However, later research has shown some criticism of this position and suggested the importance of other factors, such as length of exposure or education (e.g., Hakuta et al., 2003; Birdsong, 2014). A recent study found strong statistical evidence for native-like attainment when language acquisition began before 10–12 years (Hartshorne et al., 2018). Past research revealed mixed results as to whether earlier-exposed bilinguals have an advantage over later-exposed bilinguals when it comes to language processing. Findings by Weber-Fox and Neville (1996) suggested that there was a significant advantage for early bilinguals. These authors investigated the effect that the age of exposure (age of L2 exposure groups: 1–3, 6–7, 7–10, 11–13, and after 16) had on the processing of morphology and component distribution with Chinese–English bilinguals. They found that although delays in processing speed were only noted for the groups who were exposed to L2 English after age 11, grammaticality judgment was diminished by delays in L2 exposure as short as 1–3 years. Findings from Wartenburger et al. (2003) garnered further support for the idea of differences in language processing due to the age of onset of acquisition with Italian–German bilingual participants, differences which significantly affected the cortical representation of grammatical processes (age of L2 exposure groups: at birth vs. at or above 6 years of age). Another processing difference that has been noted between early and late bilinguals is that the latter group required higher levels of neural activation to complete L2 than L1 tasks, whereas early bilinguals showed no activation differences between their two languages (Saur et al., 2009). However, parallel lines of research have suggested that it is not the age of onset but the attained proficiency in the L2 that is a key determinant of neural processing patterns (e.g., Perani et al., 1998; Pelham and Abrams, 2014, early AOA: around 4 and 3 years, late AOA: around 11 and 10 years, respectively).

These mixed findings furthermore resonate with studies that examine the influence of the age of onset of acquisition and the attained proficiency of a second language on the possible executive functioning advantage amongst early and late bilingual groups. For example, a study by Kalia et al. (2014) controlled for a similar level of proficiency amongst the early and late bilinguals participating in measures that tested executive functioning, but found no clear advantage across measures for any group. The work of Tao et al. (2011) also examined early and late bilingual groups; however, these participants showed disparate levels of proficiency. These authors used different tasks designed to test executive functioning, in particular monitoring and inhibitory control, in a group of early, less balanced and a group of late, balanced bilinguals and found them to perform differently, with the late group showing advantages in inhibitory control. These findings led the authors to speculate that proficiency may be the decisive factor when comparing bilinguals' language processing abilities; they also point out that acquiring a language later on may be the impetuous behind enhanced control due to the need to suppress an L1 more strongly and because L2 processing is less automatized.

Numerous behavioral studies have also explored the effect that the age at which bilinguals began acquiring their languages and the attained proficiency have on language processing. Evidence from a visual world eye-tracking study examining early and late German–Turkish bilinguals speakers as well a monolingual group, revealed a similar performance between early (simultaneous) and late (around 13 years) bilingual groups; however, a performance that differed from the monolingual speakers in an L1 target-like manner (Arslan et al., 2015). On the other hand, when comparing Spanish–English early and late bilingual groups, Lai et al. (2014) found dissimilarities between equally proficient early (under age 6) and late bilinguals (age 6–15) when processing motion events in that late but not early bilinguals exhibited between-language processing differences. Nonetheless, further studies by Foucart and Frenck-Mestre (2012) and Dijkgraaf et al. (2019) found that late bilinguals were able to process the target language similar to monolinguals. Although proficiency did play a role, in that some grammatical features were more difficult for less equally balanced bilinguals to process. These findings suggest that a combination of age of onset of acquisition, proficiency, and language-specific features all influence bilingual processing.

On the whole, when examining language processing in early and late bilinguals, findings have yielded mixed results. Research has provided evidence both for and against the ability of these speakers to process their languages in a native-like manner, processing differences between both groups, and an executive functioning advantage for either group. Furthermore, the literature investigating differences between both groups, and bilinguals in general, when processing visual context during language comprehension is quite sparse. We know from the studies reviewed above that bilinguals' languages appear to be simultaneously activated in any visual and linguistic context. This activation may be the cause of a cognitive advantage—for example, flexibility when integrating concurrently presented stimuli, visuo-spatial memory, or attentional abilities—that has been found with bilinguals. We also know from studies on monolingual speakers, that visual information is quickly used to interpret language as it unfolds, and that language comprehension is affected by visual context. It remains to be seen, however, whether the language experience of bilinguals and possible resulting cognitive advantage influences how these speakers integrate rich visual information during language comprehension.

### 1.4. Current study

Previous research on monolingual speakers has revealed the strong influence of rich visual context, and a recent-event preference, during language processing. As concerns bilingual language processing, however, this line of research is currently in the early stages. The current study, therefore, aims to extend previous research and includes bilingual speakers when examining the influence of a rich visual context on language comprehension. For this purpose, we ask (a) whether visual context effects during language processing are just as strong in bilinguals as in monolinguals, or whether bilinguals' language experience results in a more effective integration of simultaneously presented visual and linguistic information which would enable anticipatory language processing; (b) whether the early or late onset of acquisition of bilinguals' languages influences the strength of visual context during language comprehension; (c) whether the previously found recent-event preference will be replicated with English linguistic stimuli that provide a clear and early tense cue.

We conducted three visual-world eye-tracking experiments with a monolingual, an early bilingual, and a late bilingual group. In these experiments, we used the same visual materials and carried out a similar procedure to that of the previous eye-tracking experiments conducted with German monolingual adult participants (i.e., Abashidze et al., 2019). However, new linguistic materials in English were created for the current study. The eye-tracking experiments were followed by an off-line memory recall test.

Our predictions in the current study were that if the recently seen action is a strong contextual cue, which might have not been diminished due to local ambiguity of the verb in the previous research,^2^ in the current study, this preference should be diminished or even eliminated in the future tense condition through an effect of the early, clearer tense cue (auxiliary verb preceding the main verb). We expect that this earlier disambiguation in English, which differs from the later disambiguation of the German syntactic structures, might result in (earlier) preferential and anticipatory looks toward targets of future events. Furthermore, if the strength of the visual cue is on par with that of the linguistic cue (as suggested by Altmann and Kamide, 2007), then we should observe a timely effect of tense cue on the selection of the corresponding target object; however, should the rich visual context cue of the recently performed event be strongly relied on by participants while interpreting the unfolding linguistic utterance, then an overall preference for looks toward the recent-event target is expected (e.g., Knoeferle et al., 2011; Abashidze et al., 2019).

In addition to the effect of the early linguistic cue, in the bilingual groups, we should see even less reliance on the recently seen event. The reduced reliance might be explained by an ability to process concurrent visual and linguistic stimuli more efficiently as a result of a possible executive functioning advantage (e.g., Blumenfeld and Marian, 2011; Bialystok, 2018). Should this indeed be the case, bilingual participants will more effectively integrate and process both visual context and linguistic cues as they unfold. As a result, bilinguals will more rapidly decrease their looks to the recent-event target upon hearing the auxiliary verb and view the future target significantly more in the future tense condition. Furthermore, these effects should become stronger in the later word regions at the cost of reducing reliance on the visual context.

As concerns differences between the early and late bilingual groups, we explore whether late bilinguals may rely more on linguistic cues, due to their processing in the later-learned language. This later onset of acquisition may lead to a stronger inhibitory control mechanism, as the need to inhibit the more dominant language would be greater, and they may therefore focus less on the visual stimuli and pay more attention to the linguistic stimuli (e.g., Tao et al., 2011). However, it is also possible that the comparable proficiency of both the early and late bilingual groups will lead to similar processing results (e.g., Kalia et al., 2014). Alternatively, the recent-event preference is robust and participants in all three groups will preferentially inspect the recent target more than the future-event target irrespective of the early tense cue of the auxiliary verb.

At the end of the eye-tracking experiments, participants completed an off-line memory test. One aim of this test was to ascertain whether the duration of looking at an event (i.e., longer time for recent events vs. shorter time for future events) would influence the ability to correctly remember the order of events within a given trial. The results of the memory test from previous studies (e.g., Abashidze et al., 2019) revealed that longer eye-gaze at recent-event targets, a likely effect of the recent-event preference, was associated with greater correct recall of events performed before, in comparison to, after sentence presentation. Accordingly, in the current study, should comprehenders interpret a language utterance about the future event quickly through the integration of a predictive language cue and linger less on the recent event, then they might show a better recall rate of the future event. By extension, should the cognitive flexibility of bilinguals allow them to process visual and linguistic cues more efficiently, then they might outperform monolinguals in the memory test. Furthermore, the visuo-spatial working memory advantage attributed to bilinguals may play a role in their being able to recall with greater accuracy the order of the events shown (e.g., Kerrigan et al., 2017; Rosselli et al., 2019).

Moreover, a recency effect has been found to play a crucial role in working memory (Zelinsky and Loschky, 2005). In this memory paradigm, the most recent items are recalled better than non-recent items (Glanzer and Cunitz, 1966). In the current study, the most recent items are the future-event target objects, those that were acted upon in the last event in chronological order. The recency effect might result in better recall of future compared to past action events; and might be found across groups.

## 2. Materials and methods

### 2.1. Participants

Experiments were conducted with one group of young monolingual (Experiment 1) and two groups of young bilingual speakers (Experiment 2 and 3, early and late bilinguals, respectively). The number of participants in Experiment 1 and 2 were 32 speakers and in Experiment 3, 28 speakers. Monolingual speakers in Experiment 1 ranged in age from 19 to 34 years [mean (*M*) = 27.72; standard deviation (*SD*) = 3.74]. Nineteen of the participants were female and thirteen were male and had, at the time of participation, 15.4 years of formal education on average. All participants were native speakers of English from Canada (14), Great Britain (3), USA (3), Australia (3), and nine without disclosure. The number of years in the country where their native language is spoken was on average 27.22. The early bilingual participants in Experiment 2 reported their L2 acquisition onset between the minimum of 0 and the maximum of 6 years (*M* = 2.6; *SD* = 1.9) of age. Their native languages were English (16), French (12), English/French (1), Vietnamese (1), Arabic (1), and Spanish (1). The ages of the participants were in the range of 19–33 years (*M* = 25.05; *SD* = 4.12). Of these participants, 20 were female and 12 were male and had 17.3 years of formal education on average. Twenty-three speakers reported their dominant language was English, while the other nine speakers reported their dominant language was French. The average number of years in the country where their native language is spoken was 22.7. The late bilingual participants in Experiment 3 reported their L2 acquisition onset between the minimum of 6 and the maximum of 12 years (*M* = 9.3; *SD* = 1.5) of age. Their native languages were French (22), German (1), Italian (1), Mandarin Chinese (1), Mohawk (1), and Spanish (1), and one without disclosure. The age of these participants ranged from 19 to 33 years (*M* = 23.32; *SD* = 3.84). Of these participants, 16 were female and 12 were male and had 16.5 years of formal education on average. All 28 of these speakers reported their dominant language to be French. The average number of years in the country where their dominant language is spoken was 21.68. All participants, mainly university students, responded to an advertisement placed on language-related Facebook groups and at different universities' campus sites in Montreal. All participants were paid $20 or they received credit points for their participation. They all had normal or corrected-to-normal vision and were unaware of the purpose of the experiment.

### 2.2. Materials and design

#### 2.2.1. Eye-tracking

**Figure 1:**
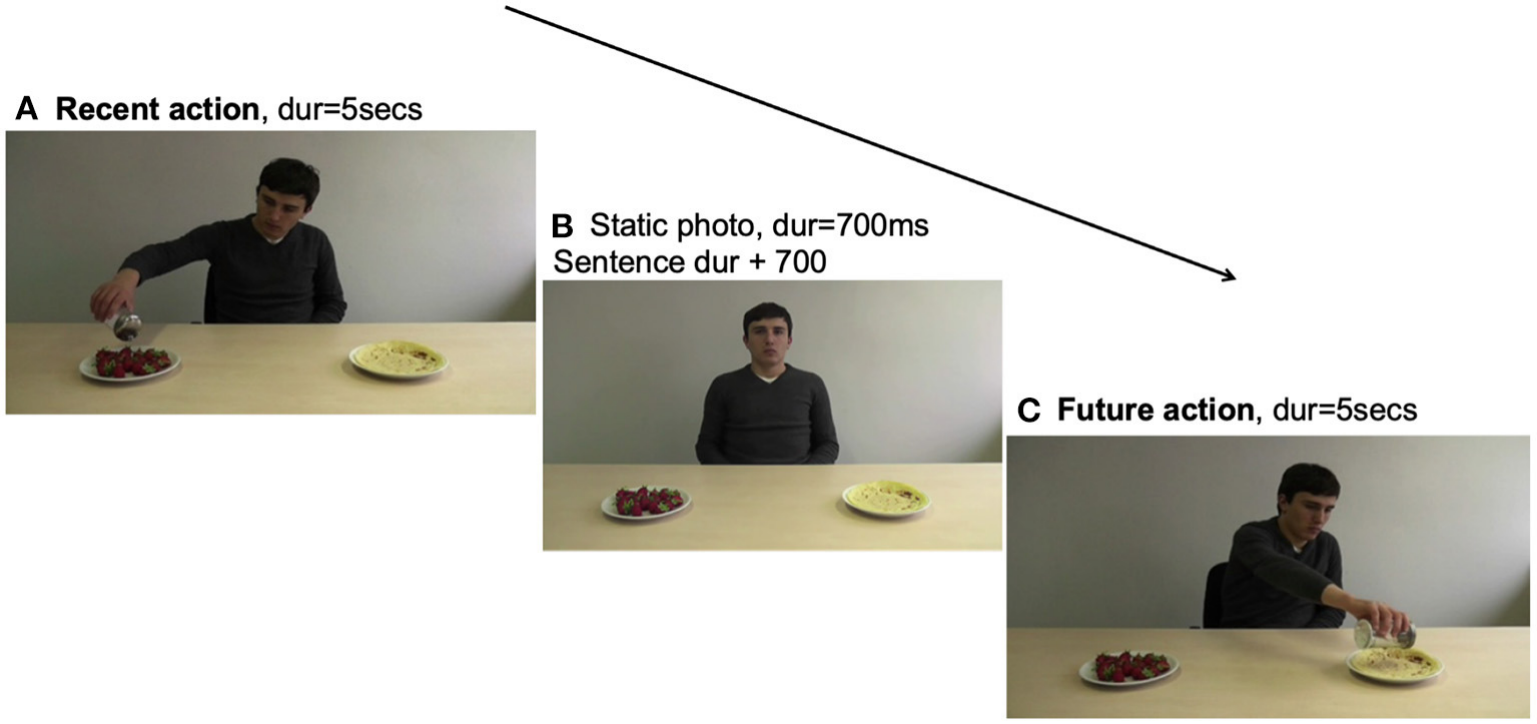
An example of the order of presentation of the visual stimuli in a typical experimental trial. **(A)** Recent action (duration = 5 s). **(B)** Static photo (duration = 700 ms) sentence duration + 700 ms. **(C)** Future action (duration = 5 s).

In all three eye-tracking experiments, the same visual stimuli were used as in the study by Abashidze et al. (2019). These visual materials were comprised of short videos (lasting an average of 5,015 ms) showing a person seated at a table on which two objects lay at an equal distance from the person (e.g., pancakes and strawberries), one to the left and one to the right side. For the linguistics materials, new sentences were recorded spoken by a male native English speaker. The structure of the critical sentences followed the same pattern (NP1-Aux-Verb-NP2, see Table 1) and the sentences referred to the events shown in the visual materials. The order of stimuli presentation in each of the 24 experimental trials was as follows: First, participants saw a video showing the person interacting with one of the objects (e.g., sweetening the strawberries, Figure 1A). Then, they viewed the last frame of the video with the person in an inactive position (Figure 1B). While this static image remained on the screen, participants heard a sentence describing either the action that had recently been performed (past tense condition, e.g., *has sweetened*; Table 1, 1b-b') or one that was yet to be performed involving the other object (future tense condition, *will sweeten*; Table 1, 1a-a'). After the utterance ended, a second video showed the person performing the same action on the other object (e.g., sweetening the pancakes, Figure 1C). In Figure 1, a typical order of presentation of the visual stimuli used in a critical item can be seen. Each experimental item consisted of two different objects (e.g., pancakes and strawberries) and four sentences (see Table 1). Furthermore, as shown in Table 1, each tense condition yielded two sentences, which ensured that each object was counterbalanced as the target of both a past and future action. The position of the objects was counterbalanced across items, so that the person interacted equally often first with the object on the right or on the left side of the table. Moreover, any visual particularities of the target objects seen after sentence utterance were evenly spread across critical conditions. We also ensured that both of the objects presented within an item afforded a compatible interaction with the verbal expression (e.g., both a pancake and a strawberry can be sugared).

**Table 1:** Example experimental sentences and conditions.

**Tense condition**	**Sentences, counterbalancing**
*1*a Future tense	The experimenter will sweeten the strawberries.
*1*a' Future tense	The experimenter will sweeten the pancakes.
*1*b Past tense	The experimenter has sweetened the strawberries.
*1*b' Past tense	The experimenter has sweetened the pancakes.

In addition to the 24 critical items, we created 40 filler items. The purpose of these filler items was to ensure that participants were exposed to a similar but varied combination of visual and linguistic stimuli. Filler items contained time adverbs indicating past and future. These were identical in all experiments. In total, participants were presented with 64 items (24 critical plus 40 filler items). The critical and filler items were combined to form four lists using a Latin square design. Each list contained every critical item in only one condition and all fillers. These lists were pseudo-randomized prior to the experiment. As a result, every participant received a uniquely randomized version of one of the four experimental lists.

#### 2.2.2. Memory test

**Figure 2:**
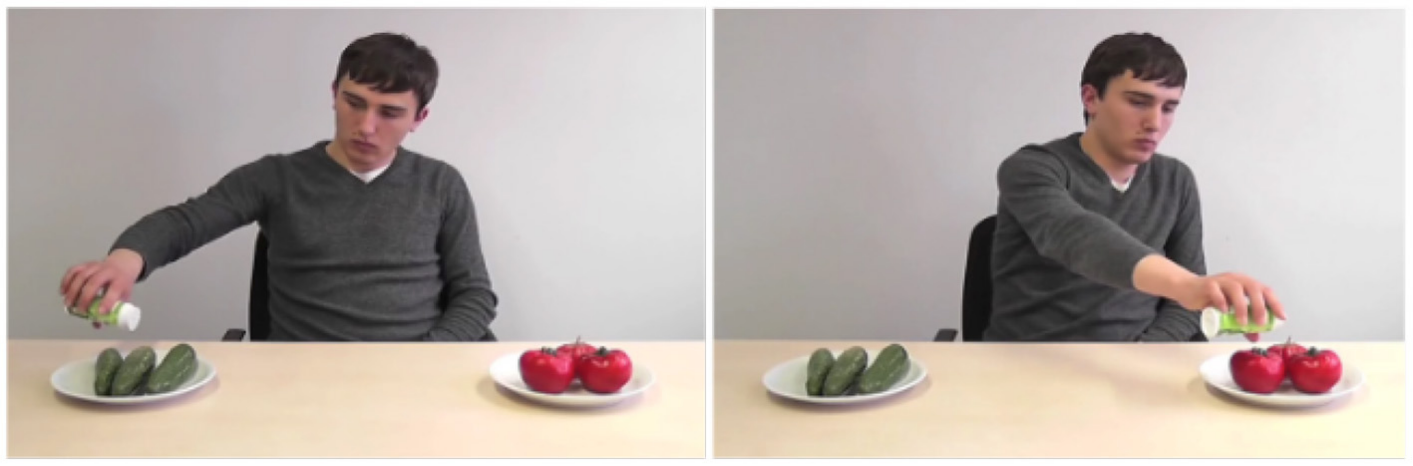
An example of a display for the memory test.

The memory test was created by using two snapshots taken from the first and second video of each experimental item. One snapshot showed the experimenter performing one action (e.g., flavoring the cucumbers) and the other snapshot showed the other action (e.g., flavoring the tomatoes). Both snapshots associated with a particular item were combined to form one image; for instance, as shown in Figure 2. In order to control the location of the paired snapshots, the snapshots were counterbalanced to the right and left positions for a given trial.

### 2.3. Procedure

For the eye-tracking session, upon arrival at the lab, participants received a printed document detailing the pertinent particulars of the experiment and were asked to confirm that they had understood it. Next, they were seated in a quiet experimental room, facing a monitor at a distance of approximately 65 cm from the screen. An eye tracker (Tobii Pro TX300, Tobii Technology AB) was used to monitor and record gaze data at 300 Hz with an average accuracy of 0.3° at optimal condition visual angle. The Tobii Studio package was used to present the videos to the participants during the experiment. All instructions provided in connection with the experiment were given in English. Participants were told that they would be shown videos presented on a screen and hear sentences relayed through a loudspeaker. The eye-tracking experiment was a slightly modified look-and-listen paradigm in which participants were instructed to understand as much as possible. They did not have any other task. The gaze of each participant was successfully calibrated prior to beginning the test proceedings using a nine-point calibration procedure, in which an attention-getter appeared in every position of a three-by-three grid of calibration points. Each trial started with a centrally located white dot which appeared against a black background for 500 ms, followed by the presentation of the first video. The last frame of the event portrayed in this video remained on the screen for 700 ms in silence, followed by the onset of the past or future tense sentences. With the offset of the sentence, the static image disappeared, and participants were shown the second action-event video. Participants could take a short break halfway through the eye-tracking experiment.

Following the eye-tracking session, each of the participants were asked to take part in a memory test. They were randomly assigned to one of the four counterbalancing lists and saw a different randomized order of the list. From this list, participants were presented with an image containing the snapshots (see Figure 2) from a single experimental items. Above the image, a written question was shown which asked either: (a) Which action was performed *before* the sentence? or (b) Which action was performed *after* the sentence?

The responses were provided by a button press. Participants were asked to press the left-hand button if they thought the left picture was correct and the right-hand button on a button box if they thought the right picture was correct. The correct answer for the left and right button press was counterbalanced for the items in each list. Upon completing the memory test, participants were debriefed. The entire duration of the experiment was approximately 45–50 minutes.

### 2.4. Analysis

#### 2.4.1. Eye-tracking data

For the eye-tracking analyses, we used the Tobii Studio software (Tobii Technology AB, Sweden) to export the fixation data. We defined two areas of interest, which comprised participants' eye-movements either toward the recent target object (e.g., the strawberries, see Figure 1) or the future target object (e.g., the pancakes, see Figure 1). Because the target images of each trial differed in size, rectangular areas of interest were defined around each image in Tobii Studio. Before running the analyses, the raw data were cleaned and checked for validity by using the eyetrackingR package (Dink and Ferguson, 2015). Any fixations shorter than 80 and longer than 1,500 ms were removed from the data. Furthermore, the trials with more than 25% of trackloss proportion were not included in the analyses. Next, we computed gaze proportions to the two target objects in each successive 50 ms time slot, starting from the onset of the sentence (the first noun phrase) until the end of the sentence (NP1-Aux-Verb-NP2). The three critical word regions were: The auxiliary region (from auxiliary onset until verb onset, mean duration = 875 ms), the verb region [from verb onset until the second noun phrase (NP2) onset, mean duration = 952 ms], and the NP2 region (from NP2 onset until NP2 offset, mean duration = 723 ms). In the first critical time window/region, fixations were counted for the analyses if they started in that time window.

**Figure 3:**
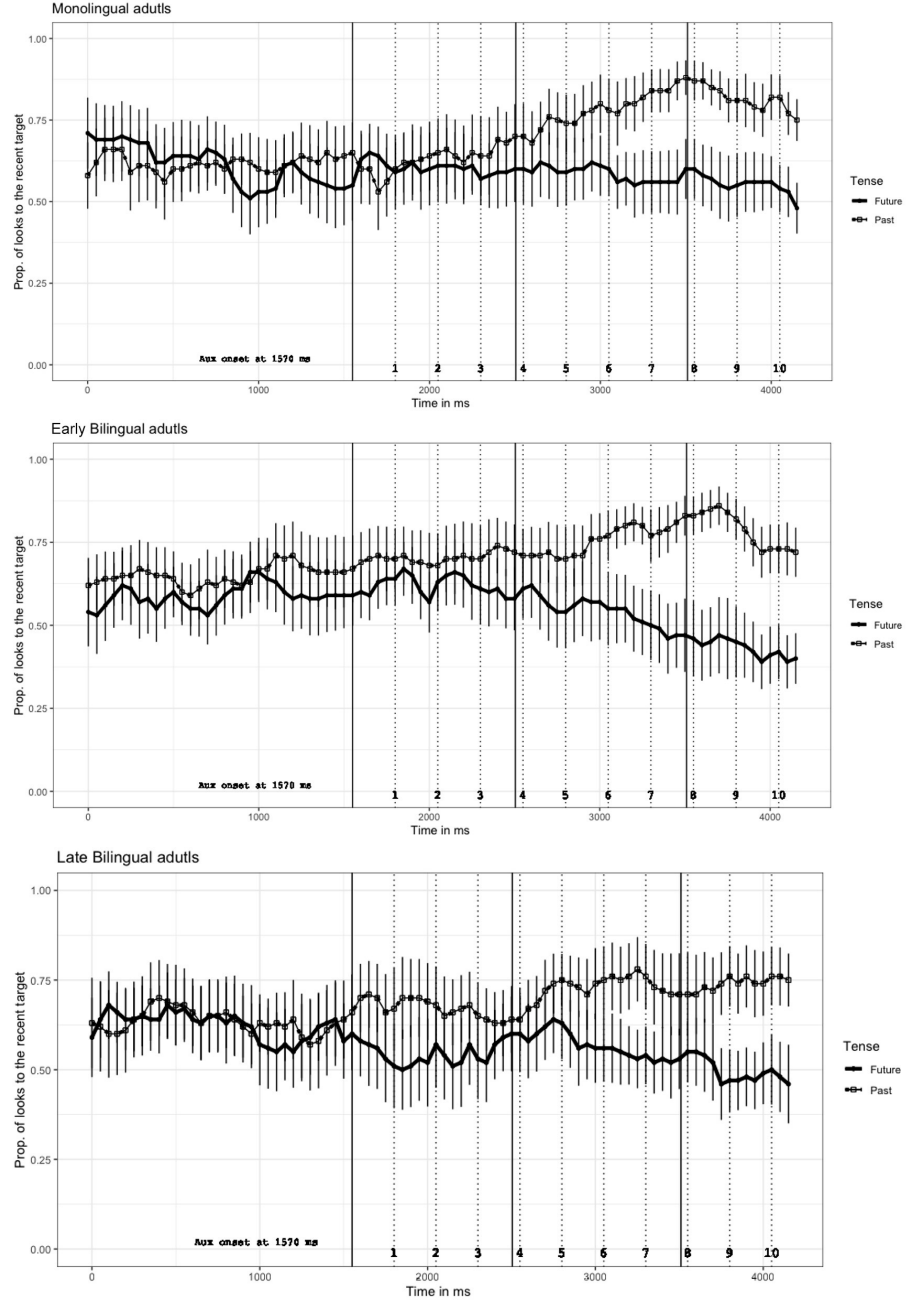
Mean proportion (with *SE*) of looks to the recent target depending on the sentence tense in Experiments 1 (monolinguals), 2, and 3 (bilinguals). Note that looks to the future target were complementary to the looks to the recent target.

As for the descriptive analyses, we created time course graphs that depict the data of the full sentences (with a mean length of 4,200 ms), which consist of the first noun phrase region, with a length of 1,570 ms, and an additional 2,630 ms after the onset of the auxiliary verb (see Figure 3). The graphs present the proportion of looks to both the recent and future target objects. The proportions of looks are presented as follows: the dotted lines indicate trials with the past tense sentences, and the solid lines indicate trials with the future tense sentences. The three vertical solid lines indicate the onset of the critical word regions. The first vertical line at 1,570 ms indicates the onset of the auxiliary verbs (*will* or *has*). The second vertical line at 2,455 ms indicates the onset of the main verb. The third vertical line at 3,457 ms indicates the onset of the second noun phrase. In this measure, a score above 0.5 reflects a preference for looking at the recent target over the future target, and a score below the 0.5 line indicates the opposite.

For the statistical analyses, we calculated the empirical logit for the looks to the recent target object, and the analysis was weighted using the procedure of Barr (2008) in all experiments. The lme4 package (version 1.1-27.1 Bates et al., 2015) was used to calculate linear mixed-effects models to assess the fixed effects of Tense and Time and their interactions, and the crossed random effect of participants and items on the empirical logit of the inspections of the target picture in all experiments.

For the between group analyses, we included the fixed effects of speakers. The contrast coding of predictors and Tense (has +1, will −1) and speakers (monoling +1, earlybiling −1)/(monoling +1, latebiling −1) and their interaction resembled those of traditional ANOVA analyses. The continuous predictor Time was created by five time bins (each 50 ms), which were examined in each 250 ms time windows (see Barr, 2008).

#### 2.4.2. Memory test

For the analyses of the post-experimental memory test results, we fitted a logistic linear mixed effect (LME) model to the binary (i.e., correct vs. incorrect) response data of these memory tests. In this model, the predicted outcome was the response and the predictors were tense (past vs. future) and target event (recent vs. future event) in all experiments. Subjects and items, with their intercepts and slopes and the intercept by slope interactions, were included in the random effects of the model. The predictors were centered by transforming the fixed effect coding into a numerical value and centering it so that it had a mean of 0 and a range of 1 (Baayen, 2008).

### 2.5. Results

#### 2.5.1. Eye-tracking results

Inspection of the target objects in the course graphs (Figure 3) reveal an overall preference for the recent target relative to the future target, as looks for both the recent and future tense conditions remained well above the chance level. This indicates that the recent target received more looks than the future target, which was confirmed by the significant intercept in all three experiments.

Monolingual participants' (Experiment 1) preferential inspection of the recent target continued throughout the auxiliary verb until the end of the NP2 region in both sentence conditions. The length of these preferential inspections occurred approximately 2,260 ms after the onset of the auxiliary verb. Despite the overall preferential looks toward the recent target, as a function of tense, participants' looks started to diverge increasingly toward the recent target in the last time bin of the auxiliary verb region. While the looks toward the recent target in the past tense condition continuously increased from this word region, the looks in the future tense condition remained mostly unchanged until the middle of the verb region. The overall gaze pattern in the NP2 region remained similar to the verb region. Only from the middle of the verb region, participants showed a slight decrease of inspections toward the recent-event target, and at the end of the NP2 region they looked more at the future target in the future compared to the past tense sentence. In other words, the looks toward the future target started to emerge only after participants heard the utterance of the second noun phrase.

Early bilingual participants (Experiment 2) similarly showed an overall looking bias toward the recent target. They, however, started to inspect the future event target in the future tense condition earlier than monolingual speakers, approximately 1,480 ms after the onset of the auxiliary verb. Moreover, as a function of tense cues, their looks began to diverge earlier in the auxiliary verb region compared to those of Experiment 1. In the middle of the auxiliary verb and at the beginning of the main verb, the two lines come closer to each other for a short period, which indicates a reduction of using the tense cue; however, this changed at the end of the verb region. From the end of the verb, participants showed a reversal gaze pattern in the future tense condition; they looked at the future target object more than the recent target object. Subsequently, the gaze pattern throughout the NP2 region shows an increasing inspection of the recent target object only in the past tense condition and an increasing inspection of the future target object only in the future tense condition. Importantly, early bilingual speakers showed anticipatory eye-movements toward the future-event target in the future tense condition. Thus, the looks toward the future-event target began 700–800 ms earlier in the early bilingual group than in the monolingual group (see Figure 3).

Late bilingual participants (Experiment 3) overall preferentially inspected the recent target. This behavior was similar to that of participants in Experiment 1 and 2. The late bilinguals, however, began to look at the future-event target in the future tense condition earlier in the NP2 region than the monolingual speakers, but later than early bilingual speakers. These looks began approximately 2,130 ms after the onset of the auxiliary verb. Furthermore, they remarkably showed the earliest decrease of looks toward the recent-event target in the future tense condition compared with the other speaker groups. That is, upon hearing the auxiliary verb, they started to decrease their looks toward the recent target object in the future tense sentence and, as expected, increase their looks toward the recent target object in the past tense sentence condition. Then, as seen in Figure 3, with the offset of the auxiliary verb, the two lines indicating inspections to either the past or future target came closer to each other only to diverge once more in the verb region until the end of the sentence. This pattern shows that looks to the future-event target in the future tense continuously increased upon hearing the main verb and from the middle of the NP2 region onward, participants looked more at the future target than at the recent target in the future tense condition.

In summary, a preference of inspections toward the recent target were shown in all three experiments; however, bilingual participants seemed to decrease their reliance on the recent target earlier than the monolingual participants during the processing of the utterance. In particular, the early bilingual groups showed a strong non-reliance on the recent-event shortly before and during the last word region. Furthermore, the late bilingual group revealed a reliable decrease in looks toward the recent-event target at the earliest stage in the sentence processing. Moreover, while monolingual participants showed a recent-event target preference in both sentence conditions, bilingual participants inspected the future-event target comparatively more often in the future tense sentence condition.

Table 2 presents the statistical results of all three experiments. The table lists the estimates (*b*) and *t*-values (*t*) for the fixed effects of the models in time windows of 250 ms between 250 and 1,750 ms from the auxiliary onset. We did not expect a reliable effect of the utterances' earliest cue in the first time window between 0 and 250 ms (see also Altmann, 2011; Abashidze et al., 2022), which was confirmed by non-significant results (*b* = −0.015, *t* = −0.108) in Experiment 1, (*b* = 3.620, *t* = 0.193) in Experiment 2, and (*b* = −1.304, *t* = −0.618) in Experiment 3.

**Table 2:** Fixed effects of the models predicting the inspections of the recent target.

	**(250–500)**		**(500–750)**		**(750–1,000)**		**(1,000–1,250)**		**(1,250–1,500)**		**(1,500–1,750)**	
	***b***	***t***	***b***	***t***	***b***	***t***	***b***	***t***	***b***	***t***	***b***	***t***
**Experiment 1**												
Intercept	0.725	2.986	0.945	3.919	9.297	3.247	8.797	3.756	1.149	4.755	1.215	6.024
Time	0.001	0.323	−0.001	−0.662	4.480	0.484	1.591	1.778	0.001	0.763	−0.001	−0.468
Tense	−0.015	−0.108	0.149	1.010	4.390	**3.111**	4.224	**3.056**	0.442	**3.481**	0.707	**5.874**
Time × Tense	0.001	0.001	0.001	1.146	1.817	0.020	3.852	0.043	0.001	1.547	0.000	0.975
**Experiment 2**												
Intercept	1.090	5.309	1.119	4.666	3.411	4.814	0.999	4.600	0.891	4.018	1.031	5.156
Time	−0.001	−0.736	0.001	0.280	−3.647	−0.466	−0.001	−1.000	0.001	0.323	−0.001	−0.173
Tense	0.245	1.958	0.316	**2.621**	3.351	**2.972**	0.0297	**2.434**	0.496	**4.200**	0.867	**7.698**
Time × Tense	0.001	0.736	−0.001	−0.207	−1.871	0.000	0.001	1.652	0.001	1.644	0.001	0.317
**Experiment 3**												
Intercept	0.735	2.733	8.002	3.311	0.725	3.358	0.845	3.547	1.223	4.916	0.964	4.277
Time	0.001	0.458	−3.661	−0.367	0.001	0.114	0.001	1.681	−0.001	−1.234	0.001	0.659
Tense	0.525	**3.589**	2.628	1.719	0.213	1.420	0.143	1.006	0.370	**2.642**	0.648	**4.750**
Time × Tense	−0.001	−0.908	1.600	0.016	−0.001	−0.397	0.001	0.881	0.001	1.180	0.001	0.302

Significant value at α =0.05, |*t*|, ≥2 are indicated in bold, in Experiments 1, 2, and 3.

In Experiment 1, the descriptive findings were confirmed by statistical analyses, as the increased looks to the recent target object after hearing the auxiliary in the past tense condition were significantly different from the looks after hearing the auxiliary in the future tense condition (*b* = 4.390, *t* = 3.111) in the third time window (750–1,000 ms). As expected, based on the descriptive results, the significance was confirmed in the following time windows as well. The analyses did not show any significance of time or an interaction between time and tense. This means that there was not a reliable increase of inspection in time in either time window (see Table 2, Experiment 1).

In Experiment 2, the descriptive findings indicated in the time course graphs were confirmed by statistical analyses. In comparison to the monolingual group, the early bilingual group showed a significant tense effect (*b* = 0.316, *t* = 2.621) in an earlier time window (500–750 ms). This finding indicates that there was a significantly early divergence of inspections toward the recent target in both tense conditions. Similar to Experiment 1, the tense effect occurred in all of the following time windows (see Table 2, Experiment 2). The analyses did not reveal any reliable effect of time and/or an interaction in this experiment either.

In Experiment 3, the reliable effect of tense was found already in the second time window as suggested in the time course graph. While the earliest tense effect in the other experiments was found in the fourth time window (Experiment 1) and in the third time window (Experiment 2), a significant tense effect in this group was already revealed in the second time window (250–500 ms), (*b* = 0.525, *t* = 3.589). This finding reveals that the late bilingual participants used the tense cue at its earliest stage and they significantly decreased their inspection of the recent target in the future tense condition. Interestingly, the statistical analyses in the following three time windows did not show a reliable significant effect of the tense (see Table 2, Experiment 3). The strength of the earliest tense cue in this group does not seem to be stable. However, the effect became significant again from the middle of the verb region and it lasted throughout the sentence. Similar to Experiment 1 and 2, Experiment 3 did not show any significance of time and/or an interaction in the preferential inspections.

Crucially, a comparison between Experiment 1 and Experiment 2 as well as a comparison between Experiment 1 and Experiment 3 did not reveal any reliable effects of the speaker groups in the compared time windows. The gaze pattern of these groups toward the recent- and future-event target did not differ significantly in either condition.

#### 2.5.2. Memory test results

**Figure 4:**
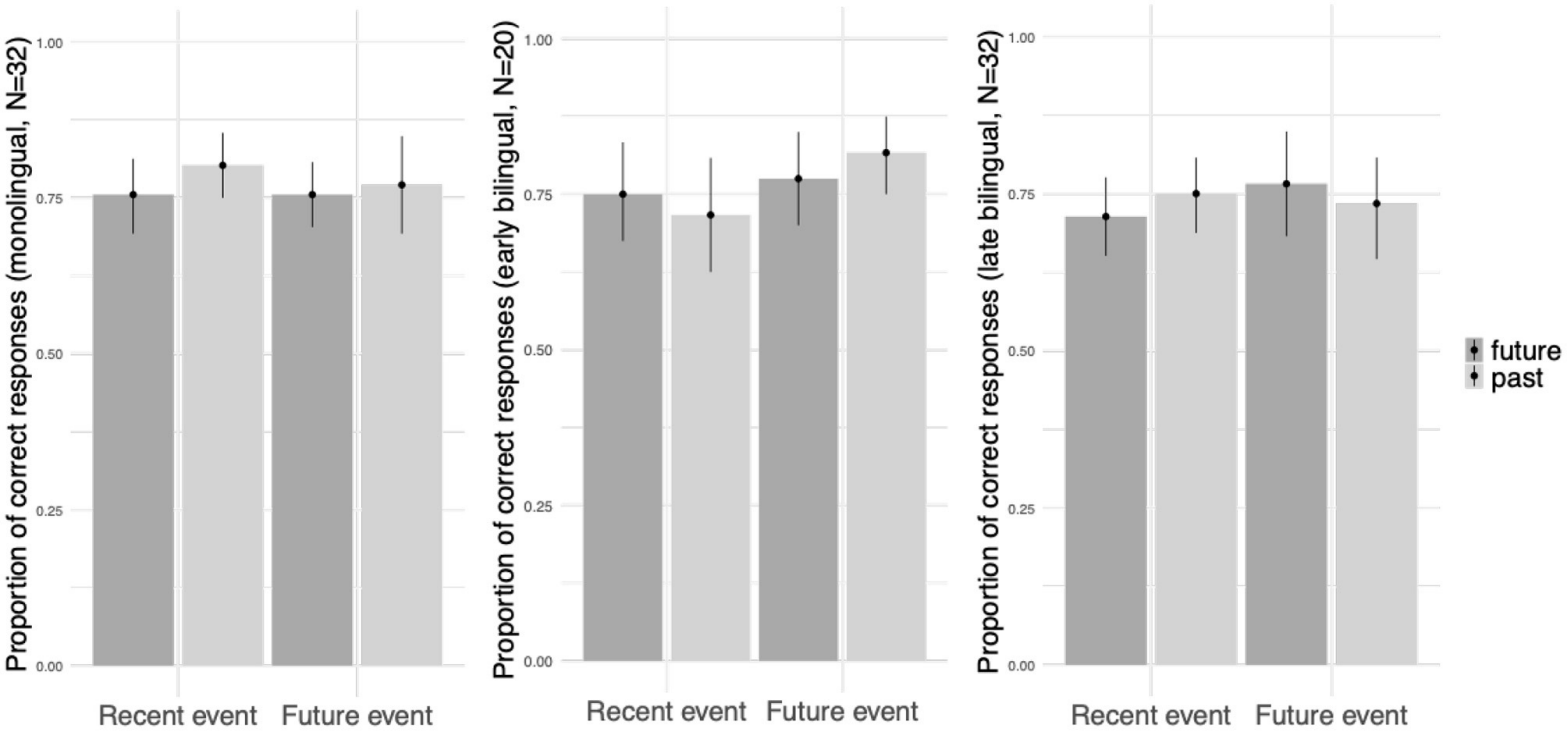
Proportion mean of correct answers as a function of event and tense in Experiments 1, 2, and 3 (with error bars plotting the standard error of the mean).

The results of the memory tests of all three experiments show the accuracy averaged by conditions (by participants) and are depicted in Figure 4. Monolingual participants (Experiment 1) correctly answered 0.77 of the questions. They were overall slightly more accurate in recalling the recent compared to the future event (0.78 vs. 0.76). They also were slightly more accurate in recalling the recent action events in the past tense vs. the future tense condition (0.80 vs. 0.75), while the future event in both tense conditions was equally recalled. Early bilingual speakers (Experiment 2) correctly answered 0.76 of the questions. They were overall better in recalling the future events than the recent events (0.79 vs. 0.73). However, unlike in Experiment 1, the sentence tense information was not used to support memory recall across conditions. Late bilingual speakers (Experiment 3) correctly answered 0.74 of the questions. As in the early bilingual group, the late bilingual speakers were overall slightly more accurate in recognizing the future event over the recent event (0.75 vs. 0.73). Furthermore, they recalled the future event better in the future tense compared to the past tense condition (0.77 vs. 0.73). The statistical analyses did not reveal any reliable differences in recalling the recent vs. future events in all three experiments (all *p*s > 0.16).

The results of the memory test, as far as the effect of the recent-event preference is concerned, support the findings of the eye-tracking experiments to a certain extent. The eye-tracking results show a high preference for the recent-event target, which decreased earlier for bilingual speakers. Comparably, monolingual participants recalled the recent event better than the future event, similar to the previous findings with German monolinguals (e.g., Abashidze et al., 2019, Experiment 1). Both bilingual groups were better able to recall the future event compared to the recent event. Just as with their eye-gaze data, bilingual participants' memory test data also revealed a decreased reliance on the recent event.

## 3. Discussion

In three visual-world eye-tracking experiments and post-experimental memory tests, we investigated the integration of visual context in monolingual and bilingual language processing. Prior research on monolingual language processing has documented the robust influence of visual context in monolingual speakers (e.g., Knoeferle and Crocker, 2007; Knoeferle et al., 2011; Abashidze and Chambers, 2016; Abashidze, 2017), even in studies where immediate gaze cues or frequency distributions might have countered this preference (e.g., Abashidze et al., 2019; Abashidze and Knoeferle, 2021). With bilinguals, research on language processing within a visual-world paradigm has greatly focused on the degree of activation of a bilinguals' languages as a result of the visual context (e.g., Chabal et al., 2015; Hartsuiker, 2015) and the sensitivity toward simultaneously presented language modalities (Marian, 2009; Shook and Marian, 2012). These studies have revealed the simultaneous activation of a bilinguals' languages—which would consequently require the inhibition of the non-target language—and led numerous researchers to suggest that bilinguals may be advantaged when performing tasks that require cognitive control (e.g., Bialystok and Martin, 2004; Blumenfeld and Marian, 2011; Bartolotti and Marian, 2012; Bialystok, 2015). Other studies have focused on how the degree of billigualism affects cognitive functioning and have yielded mixed results (e.g., Tao et al., 2011; Kalia et al., 2014). Similarly, studies examining language processing comparing early and late bilinguals do not always show clear differences between the two groups (e.g., Weber-Fox and Neville, 1996; Wartenburger et al., 2003; Saur et al., 2009; Lai et al., 2014; Pelham and Abrams, 2014; Arslan et al., 2015). Thus, it is still largely unknown how different groups of bilinguals process linguistic information when it is accompanied by a rich visual context.

With the current study, we built upon previous findings and extended the areas of inquiry to encompass a monolingual and an early and a late bilingual group. Within the experimental setup, visual context information was in the form of an action event which showed a person performing an action on an object (such as sweetening strawberries or pancakes, see Figures 1A, C). After viewing this action, participants heard a sentence in English (NP1-AUX-V-NP2) that described the action either as having been recently performed or as yet to be performed (see Table 1). After the presentation of the linguistic utterance, participants viewed another action event involving the other object in the scene. The aim of this research was to examine (a) whether the cognitive effects of bilinguals' language experience facilitate a decreased reliance on rich visual context during sentence processing and result in early anticipatory eye-movements toward the plausible future event target in the future tense condition; (b) whether early and late bilingual speakers differ in their integration of visual and linguistic cues during language comprehension; (c) whether the timing of early linguistic cues affects the recent-event preference and guides participants' attention sooner toward a plausible future-event target.

We predicted that the strength of the recent visual context, which has been shown to guide a comprehender's attention during language processing, would be diminished. The quicker attenuation of this bias would be an effect of the early linguistic cue (auxiliary verb preceding the main verb) in English. This earlier cue differed from the locally ambiguous sentence components of previous research (e.g., Knoeferle et al., 2011; Abashidze et al., 2019). Such an effect would support findings that show the rapid integration of grammatical cues in sentence processing (e.g., Altmann and Kamide, 2007; Chambers and San Juan, 2008).

Furthermore, in addition to the use of the early linguistic cue, bilingual speakers should show even less reliance on the recently portrayed rich visual context as a consequence of an advantage in executive function. This advantage would facilitate being able to switch focus between simultaneously presented stimuli during language processing and point toward stronger attention and cognitive control abilities, as suggested by other researchers (e.g., Blumenfeld and Marian, 2011; Chabal et al., 2015; Bialystok, 2018). As for differences between the early and late bilingual groups, we explored whether late bilinguals may rely more on linguistic cues, due to their processing in the less dominant, later-learned language, which may result in a stronger inhibition of non-L2 stimuli, in line with the stronger inhibitor effects suggested, for example, by Tao et al. (2011). Moreover, these effects should become stronger in the later word regions at the cost of reducing reliance on the visual context.

Analyses of the eye-movement data in all three experiments revealed that participants overall preferentially looked at the recently acted upon target. At first glance, these preferential gaze patterns replicate the findings of the recent-event preference in previous studies (e.g., Knoeferle et al., 2011; Abashidze, 2017). The monolingual participants of our Experiment 1 (see Figure 3, monolingual adults) inspected the recent-event target in both sentence conditions throughout the sentence; and only at the end of the sentence, similar to the previous research of Knoeferle et al. (2011), Experiment 2, they did start to inspect the future-event target in the future tense condition. However, the bilingual participants in current Experiments 2 and 3 exhibited a different gaze pattern. In contrast to the monolingual participants, the bilingual groups inspected the future target object more than the recent target object in the future tense condition during the last word region and even earlier in the early bilingual group. In other words, they started to anticipate the future target object earlier than their monolingual participants but later than in findings from other studies that applied frequency and gaze cue distribution with German monolinguals (e.g., Abashidze et al., 2019; Abashidze and Knoeferle, 2021).

These findings of an overall recent-event preference throughout the sentence utterance (more in Experiment 1 than in Experiments 2 and 3) support accounts that prioritize the grounding of verb reference in a recent action over utilizing tense cues for early anticipation of another plausible action event (e.g., Knoeferle and Crocker, 2007; Knoeferle et al., 2011; Abashidze et al., 2019). It should, however, be noted that the early linguistic cue in the current studies affected listeners' visual activity in the future tense condition compared to the previous studies (in German with a 50–50% past/futuric present frequency distribution) by decreasing the bias of looking toward the recent target. In contrast to these previous studies, participants in the current Experiment 1 made fewer eye-movements toward the recent target as early as the auxiliary verb region and, as an effect of the tense cue, started to significantly decrease their inspections of the recent event in the future condition compared with the past condition.

With respect to the early linguistic cue, in Experiment 1, the effect was less pronounced in the first 700 ms when participants heard the auxiliary verb irrespective of its tense. Thus, the looks toward the recent target did not differ between the future and past auxiliary verbs. Monolinguals began to diverge their looks toward the recent event in the past and future sentence conditions only at the end of the first word region. This gaze pattern of changing the bias toward the recent target after the first critical word in the sentence is in line with the finding of Abashidze et al. (2019) Experiment 2, (with the frequency distribution of 75–25%). Moreover, the significance of the tense occurred in the middle of the main verb region, which is earlier than the tense effect that was found in the second experiment of Abashidze et al. (2011); Knoeferle et al. (2011, with 50–50% of frequency) in which the recent and future event as well as the futuric present and past tense sentences were equally often presented in German similar to the current study. Similarly, the effect of the verb agrees with other findings that showed an earlier influence of the verb cue in the anticipatory process but not as early as in the past studies (e.g., Altmann and Kamide, 1999; Kamide et al., 2003). We should, however, note that the previous studies by Altmann and colleagues utilized static images of a plausible action (e.g., Altmann and Kamide, 2007). One might then consider to what degree the richness of the visual context influences language processing and take this into account when comparing data collected using static images compared to real-world performed actions (e.g., see Saryazdi et al., 2018, for a discussion). Overall, the early linguistic cue affected the strength of the recent-event preference but it did not override this overall preference throughout the sentence utterance.

The aim of Experiment 2 was to discover how bilingual speakers interact with cues from a rich visual context in their anticipatory language processing. Bilingual gaze behavior data, compared to that of monolinguals, show the earlier integration of contextual cues, which points to the possibility that early bilinguals showed a greater flexibility when encountering numerous simultaneously presented stimuli, as they were able to more quickly integrate the tense cue provided by the linguistic stimuli (see Hartsuiker, 2015, for a discussion of using visual cues in bilingual language selection). This finding might be explained by studies on executive functioning in bilinguals which provided evidence for a superior monitoring advantage for bilinguals over monolinguals (e.g., Blumenfeld and Marian, 2007; Bialystok, 2018). Similar to the monolingual group of Experiment 1, early bilinguals looked more toward the recently performed action across both tense conditions, which shows that the visual cue still held precedence and captured participants' attention. However, this attention did not have as strong a hold on early bilingual participants. Gaze data show that the divergence of looks that began at 300 ms and then again around 600 ms after auxiliary verb onset became incrementally more pronounced throughout the sentence, with the notable result that the looks to the future target object in the future tense condition overrode the looks to the recent target object shortly before participants heard the NP2. These anticipatory inspections of the future target object occurred 750 ms earlier than with their monolingual peers. Thus, the early bilinguals not only showed an increase of correct looks toward the future target but these looks also reflected the effect of an early tense cue. Moreover, the tense significance was found one time-window (250 ms) earlier in the bilingual than in the monolingual group (see Table 2, Experiments 1 and 2).

In Experiment 3, we find gaze patterns that again point to differences between monolingual and bilingual language processing but also to differences between the early and late bilingual groups. The greatest divergence between looks toward either target object as a result of tense cue introduced through the auxiliary verb were found amongst late bilinguals as early as 300–600 ms post auxiliary verb onset; and this effect was statistically confirmed to be significant (see Table 2). This decreased reliance on the visual cue might be explained by the greater inhibitory control attributed to late bilingual speakers as a result of their having learned the language later and the need to inhibit the L1 was necessary to a stronger degree (Tao et al., 2011), causing them to pay more attention to the incoming linguistic information of their L2. This focus on the linguistic over visual cues might further be explained by studies showing a greater cognitive load for L2 processing with late bilingual speakers (Wartenburger et al., 2003; Saur et al., 2009), by which the linguistic cues elicited a stronger demand for their attention. However, interestingly, with the onset of the main verb, looks to the recently performed event again became more pronounced, so it is possible that listeners, although showing a bias to select the correct target upon hearing the linguistic cue, still chose to verify the incoming information by looking toward the recently performed past action during the presentation of the main verb, supporting previous findings that point toward the grounding of the verb in the visual context (Knoeferle et al., 2011; Abashidze et al., 2019, experiments with monolinguals). Nonetheless, similar to the early bilingual participants of Experiment 2, late bilinguals began to gradually decrease their looks to the recent-event target object in the future tense condition with more looks to the correct future target following the onset of the NP2. This gaze pattern supports findings showing that high proficiency leads to similar language processing patterns (Arslan et al., 2015) and cognitive flexibility (Kalia et al., 2014) across early and late bilinguals speakers.

Although the differences between monolingual and bilingual speakers did not reach statistical significance, bilinguals, perhaps due to their cognitive flexibility (e.g., Blumenfeld and Marian, 2011; Kazemeini and Fadardi, 2016; Yang and Yang, 2017; Ikizer and Raḿırez-Esparza, 2018), appear to outperform monolinguals at reducing the reliance on the recently viewed action event—which, when considering the equal distribution of past and future action events should not have been assigned greater relevance—while processing the linguistic utterances. The results of the current study may reflect bilinguals' earlier return to linguistic tense cues compared to monolinguals, after successfully inhibiting the preceding visual context information. In other words, it is possible that bilinguals show less overall persistence with previous (currently irrelevant) cues than their monolingual peers. The capability of detaching from irrelevant sources more promptly might be a decisive feature of bilingual language processing. These findings show that the recent-event preference is not absolute, as it was affected to a certain extent (in particular in the bilingual groups) at the earliest stages of sentence processing when listeners received temporal linguistic cues pointing toward a future event. This pattern shows that bilinguals were able to switch attention to the more relevant linguistic information and inhibit the stronger visual cues and does not support research showing no bilingual advantage in executive functioning (e.g., Wodniecka et al., 2010; Kousaie et al., 2014; Antón et al., 2019).

In summary, this relative rapid integration of linguistic cues partially supports the incremental processing accounts of numerous previous studies (e.g., Kamide et al., 2003; Altmann and Kamide, 2007). Furthermore, this ability to quickly interpret the information contained in sentence utterances and predict the future target is in line with research showing that highly proficient bilinguals perform on par with monolingual speakers (Foucart and Frenck-Mestre, 2012) and that late bilinguals are adept at effectively processing linguistics cues situated in visual contexts (Dijkgraaf et al., 2019). This implies that the bilinguals might rely as strongly on linguistic as visual cues when processing language, a prediction can be tested in future research extensively by combining visual and linguistic cues in different experimental setups.

The post-experimental memory test following the eye-tracking experiments yielded mixed results. On the one hand, the findings were in line with the recent-event preference patterns found in the eye-tracking experiments, such that in memory test 1, monolingual participants recalled the recent event in the past tense sentence condition better than in the future tense sentence condition; however, this recall was not significantly reliable (similar findings were reported in the previous studies by Abashidze et al., 2019). In contrast, early bilingual participants in memory test 2 as well as late bilingual participants in memory test 3 exhibited an overall better recall of the future event compared to the recent event. Moreover, the late bilingual participants recalled the future event better in the future tense condition than in the past tense condition; however, this effect was not significantly reliable.

The better recognition of visual stimuli in the recent condition with monolingual participants could be explained by the strong preference for recent events and hearing utterances referring to those events, evoking more in-depth cognitive processing and increased attention to the stimuli, thus benefiting the later recall of visual event information (see also O'Brien and Raymond, 2012). Hence, we see that processed content (for example, tense cues or viewed action events) has effects on the ability to remember linguistic or event information. The implications of these effects underline the functional significance of the recent-event preference. Whereas a possible future action may not be verified until it unfolds, an action performed prior to the presentation of relevant linguistic information can be immediately verified (i.e., MacFarlane, 2003). This instant possibility to establish the veracity of a past event may leave deeper memory traces and thereby enable a more efficient memory recall.

The better recall of the future-event target in the bilingual groups is in line with other findings that show that the most recent stimuli are recalled better than the non-recent stimuli (see also Glanzer and Cunitz, 1966; Zelinsky and Loschky, 2005). Additionally, the findings in our study might be in relation to the cognitive flexibility found with bilinguals. The fact that in the eye-tracking experiment, the early bilinguals were able to use the linguistic cues to predict the future target and therefore spent more time looking at the future event in the eye-tracking experiments might have had the effect that they were better able to recall the future event with comparably high accuracy in the memory test. This pattern is furthermore reflected, albeit to a lesser degree, in the recall of future events found with the late bilingual speakers. Moreover, the differences in performance in the memory tests between the bilingual and monolingual groups may be a result of the advantage found with bilinguals when performing visuo-spatial working memory tasks (e.g., Kerrigan et al., 2017; Rosselli et al., 2019).

## 4. Conclusion

The present findings showed that the linguistic tense cues clearly modulated the recent-event preference earlier in the current study compared to, for example, the futuric present tense (in German) using the same frequency manipulation. This modulation was further supported by the early significance of the tense cue in the current study. Nonetheless, when speaking to the robustness of the recent-event preference, even the early tense cue at the auxiliary verb followed by the main verb did not immediately eliminate this bias. As in other studies, participants overall preferentially inspected the recent-event target. However, importantly, the bilingual speakers showed more cognitive flexibility when processing the concurrent linguistic and rich visual cues. They showed less bias toward the recent-event target and reduced their inspections toward the recent target in the future condition earlier than the monolinguals. Moreover, the data from the early bilingual group revealed a reversed eye-gaze pattern shortly before the last word region, as they inspected the future-event target more in the future than the past tense condition. This pattern reveals that early bilinguals were able to anticipate the future target before it was mentioned. In contrast to the early bilingual group, late bilinguals' did not show anticipatory eye-movements; nonetheless, they were able to use the linguistic cues sooner than the monolinguals. This greater flexibility in the integration of visual context and language comprehension found with the bilingual speakers might have been a result of bilinguals' language experience and the related executive functioning advantages. The interpretation of the results of the current experiments are subject to certain limitations. In this regard, we address the heterogeneity of the additional languages spoken by the participants, which may have influenced sentence processing. However, we expected that the high proficiency in the target language and the balanced use of bilingual participants' languages would minimize the influence of other languages and provide sufficient basis for testing the effect of bilingualism on language processing within a rich visual context.

The accuracy of recalling visual stimuli in the post-experimental memory test 1 in Experiment 1 suggests that the early linguistic cue did not influence participants' short-term memory. In memory tests 2 and 3 of Experiments 2 and 3, by contrast, tense cues seemed to have had some effects in the sense that they influenced memory recall and there was slightly better accuracy in recalling future events. Overall, the memory test results suggest the need for further research assessing the functional contribution of this attentional preference in the later recall of tense information or visual events from recent memory. Furthermore, the non-significant differences between the groups can be tested in other experimental setups, in which, for instance, another powerful cue such as actor gaze cues can be introduced to see whether or not the cognitive flexibility of bilingual speakers results in their significantly outperforming monolingual speakers when processing language within a rich multimodal context.

## Data availability statement

The raw data supporting the conclusions of this article will be made available by the authors, without undue reservation.

## Ethics statement

The studies involving human participants were reviewed and approved by Ethics Committee at Concordia University. The participants provided their written informed consent to participate in this study. Written informed consent was obtained from the individual(s) for the publication of any potentially identifiable images or data included in this article.

## Author contributions

DA: conceptualization, data curation, formal analysis, investigation, methodology, project administration, funding acquisition, writing—original draft preparation, and writing—review and editing. AS: writing—original draft preparation and writing—review. PT: conceptualization, funding acquisition, and writing—review and editing. JM: software, project administration, and writing—review and editing. All authors contributed to the article and approved the submitted version.

## Footnotes

^1^For a review on reading studies and the processing of words in a sentence context with bilinguals see Assche et al. (2012).

^2^As previously mentioned, the studies conducted in German used simple past and futuric present tense verbs. To ensure that the futuric present tense was a good cue toward the future event, a norming test was carried out in this study (Abashidze et al., 2019, p. 4–5).
