# Supplementary material for: Integration of visual context in early and late bilingual language processing: evidence from eye-tracking
Source: Front Psychol. 2023 Apr 26;14:1113688. doi: 10.3389/fpsyg.2023.1113688 (PMC10171561; doi:10.3389/fpsyg.2023.1113688)
Supplement: Supplementary file 1 [file Data_Sheet_1.pdf]

The visual materials were comprised of two short videos, which were shown before and after participants heard the experimental sentences. The presented images show the final frame from the first video of the person in an inactive position looking ahead seated at the table on which were two objects. While participants viewed this image, they listened to a sentence that referred either to the recently performed action on one of the objects shown in the first video or to an equally plausible future action involving the other object shown in the second video.

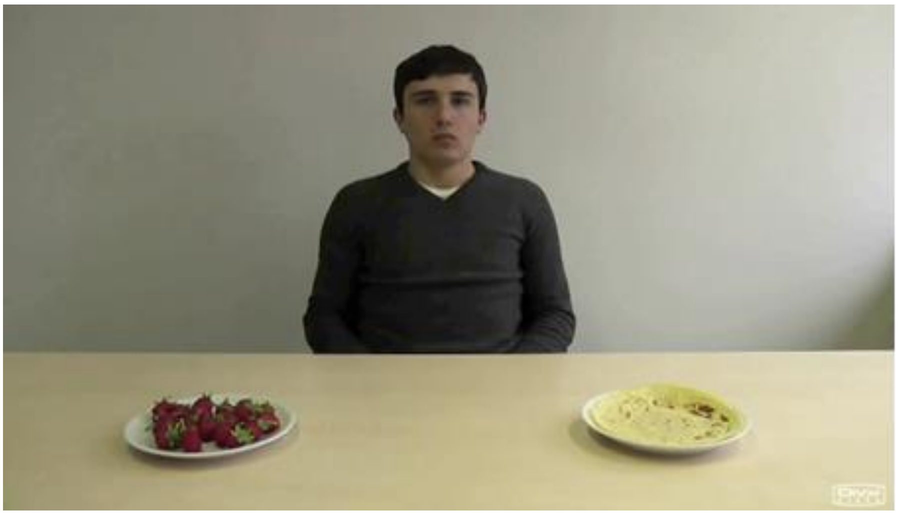

Item 1

1_1a. The experimenter will sweeten the strawberries.

1_1b. The experimenter will sweeten the pancakes.

1_2a. The experimenter has sweetened the strawberries.

1_2b. The experimenter has sweetened the pancakes.

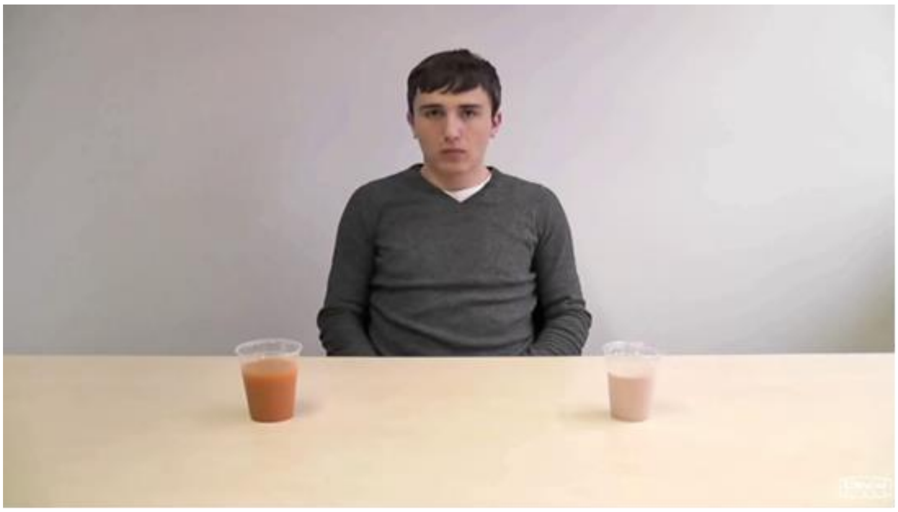

Item 2

2_1a. The experimenter will prepare the milkshake.

2_1b. The experimenter will prepare the cocktail.

2_2a. The experimenter has prepared the milkshake.

2_2b. The experimenter has prepared the cocktail.

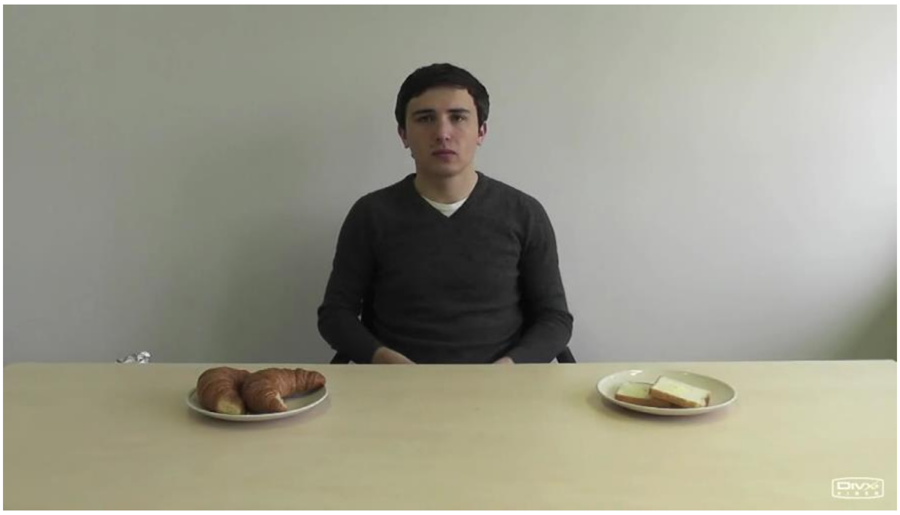

Item 3

3_1a. The experimenter will butter the croissants.

3_1b. The experimenter will butter the bread slices.

3_2a. The experimenter has buttered the croissants.

3_2b. The experimenter has buttered the bread slices.

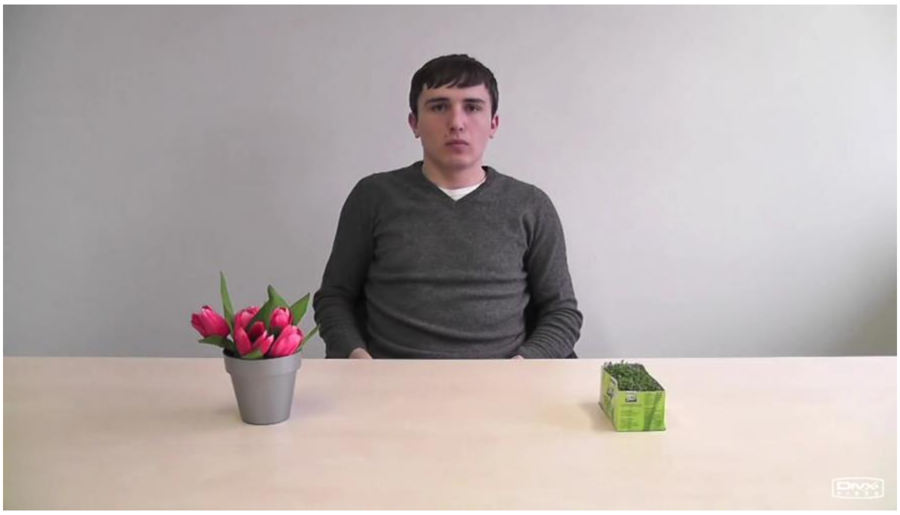

Item 4

4_1a. The experimenter will water the sprouts.

4_1b. The experimenter will water the tulips.

4_2a. The experimenter has watered the sprouts.

4_2b. The experimenter has watered the tulips.

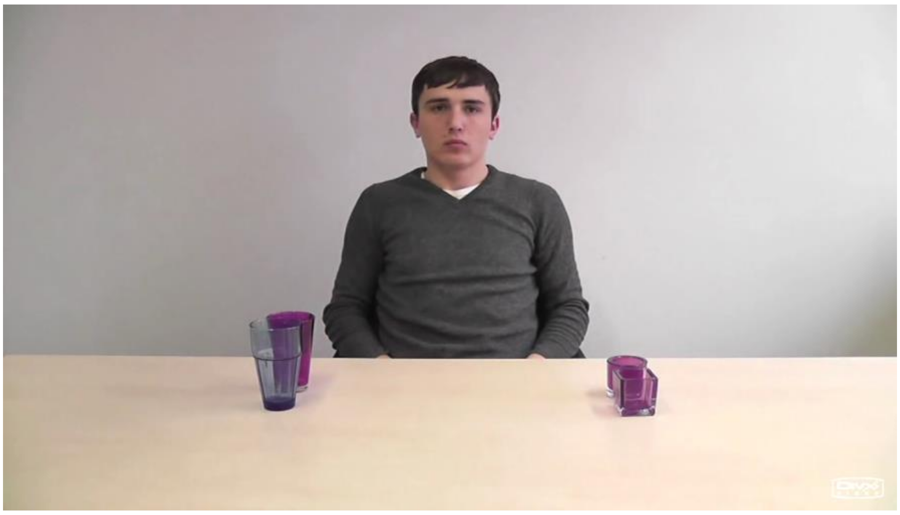

Item 5

5_1a. The experimenter will polish the candle holders.

5_1b. The experimenter will polish the glasses.

5_2a. The experimenter has polished the candle holders.

5_2b. The experimenter has polished the glasses.

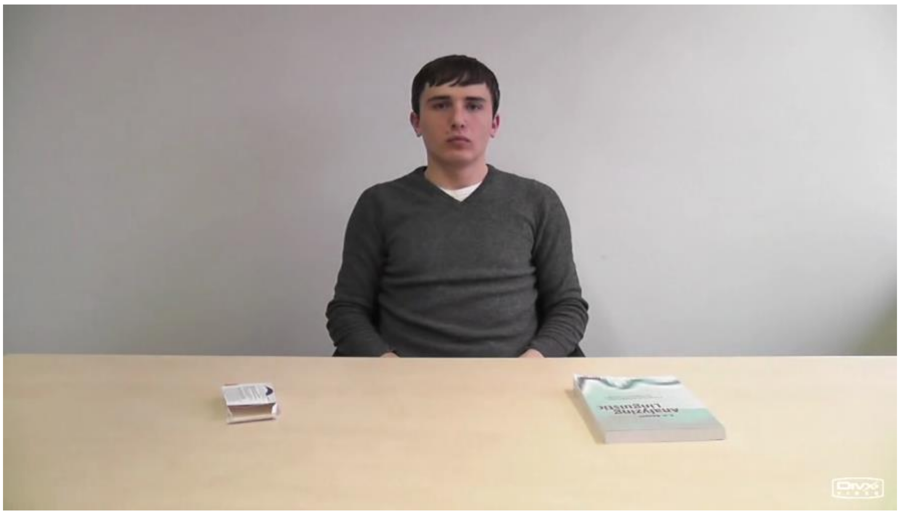

Item 6

6_1a. The experimenter will read the manual.

6_1b. The experimenter will read the book title.

6_2a. The experimenter has read the manual.

6_2b. The experimenter has read the book title.

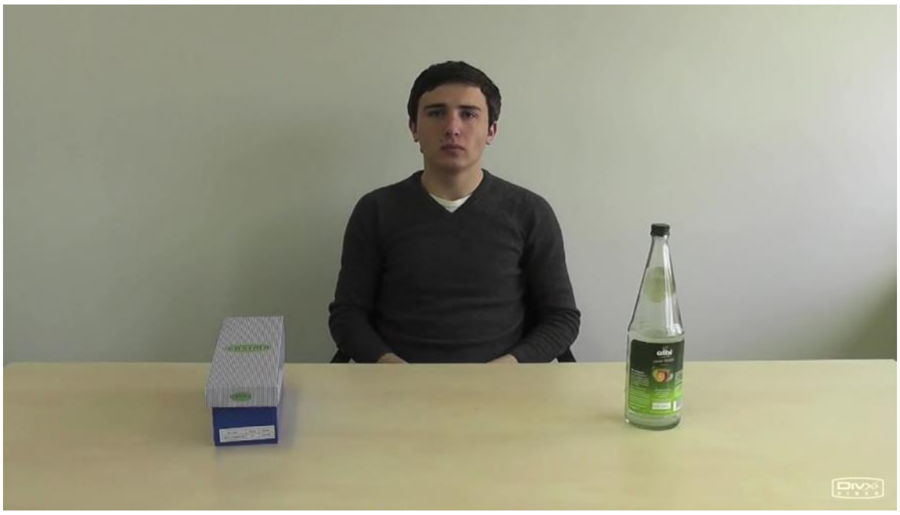

Item 7

7_1a. The experimenter will open the juice bottle.

7_1b. The experimenter will open the shoebox.

7_2a. The experimenter has opened the juice bottle.

7_2b. The experimenter has opened the shoebox.

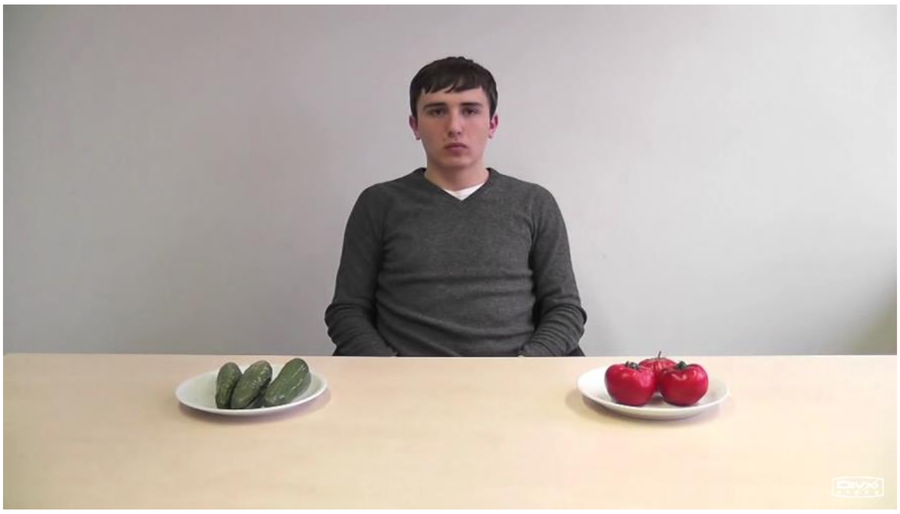

Item 8

8_1a. The experimenter will season the cucumbers.

8_1b. The experimenter will season the tomatoes.

8_2a. The experimenter has seasoned the cucumbers.

8_2b. The experimenter has seasoned the tomatoes.

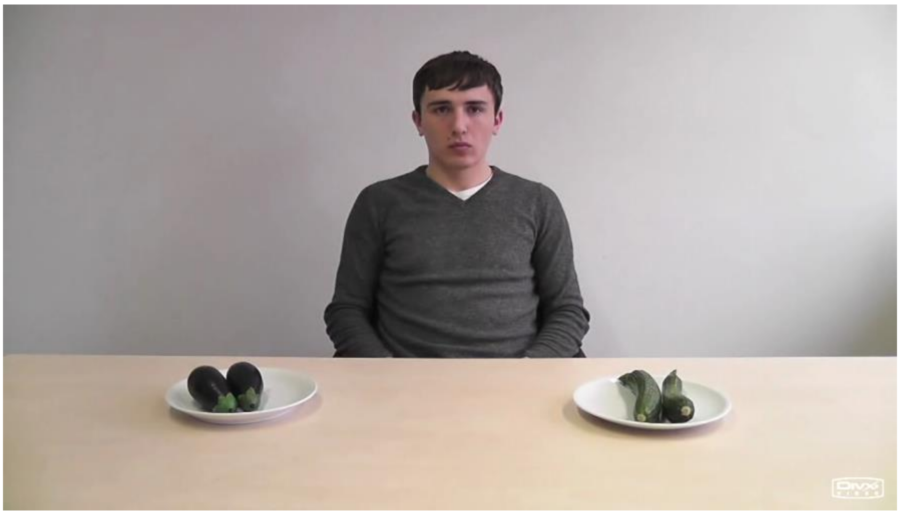

Item 9

9_1a. The experimenter will salt the eggplants.

9_1b. The experimenter will salt the zucchinis.

9_2a. The experimenter has salted the eggplants.

9_2b. The experimenter has salted the zucchinis.

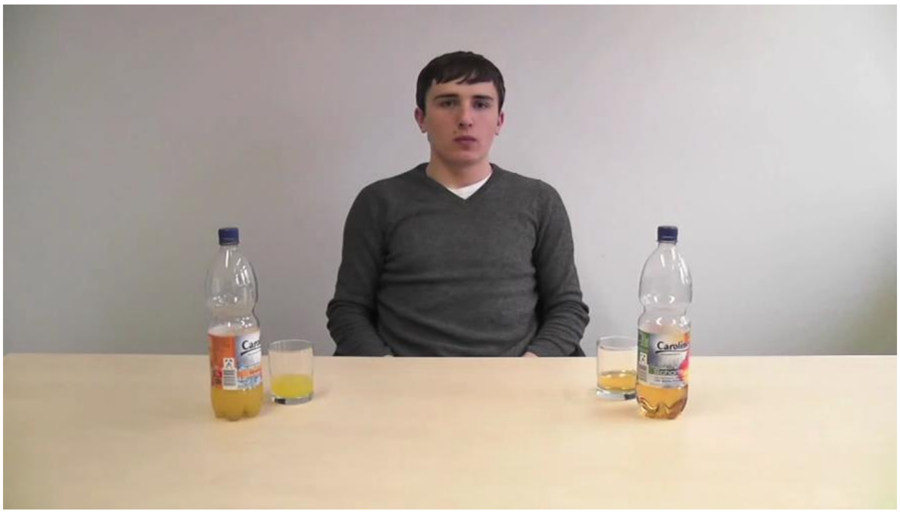

Item 10

10_1a. The experimenter will drink the apple juice.

10_1b. The experimenter will drink the lemonade.

10_2a. The experimenter has drunk the apple juice.

10_2b. The experimenter has drunk the lemonade.

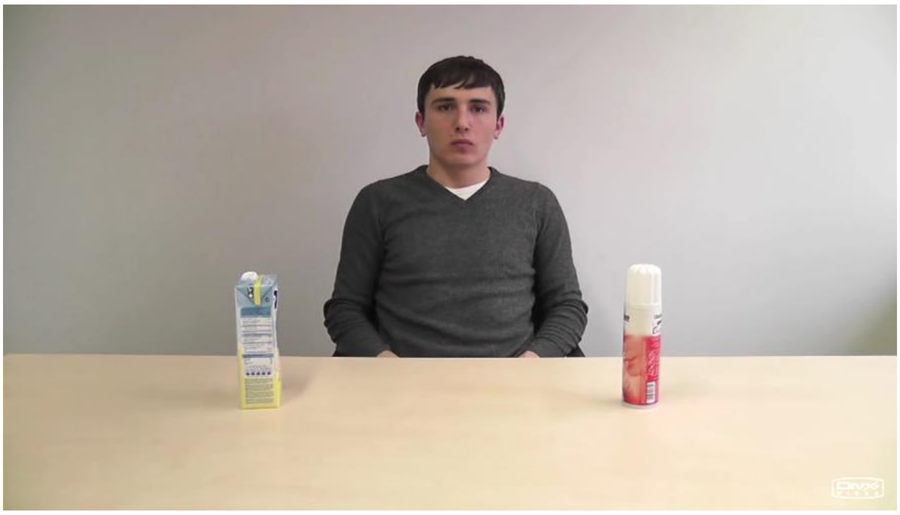

Item 11

11_1a. The experimenter will shake the soya milk.

11_1b. The experimenter will shake the whipped cream.

11_2a. The experimenter has shaken the soya milk.

11_2b. The experimenter has shaken the whipped cream.

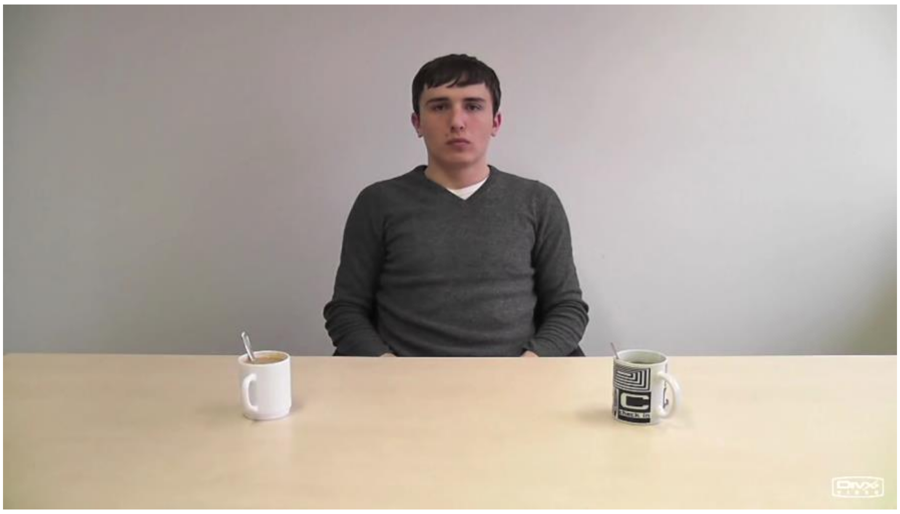

Item 12

12_1a. The experimenter will stir the herbal tea.

12_1b. The experimenter will stir the latte.

12_2a. The experimenter has stirred the herbal tea.

12_2b. The experimenter has stirred the latte.

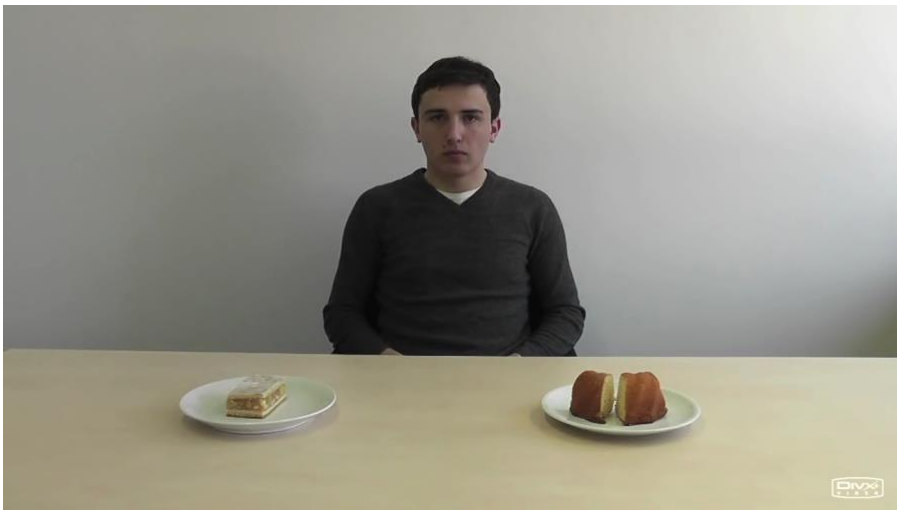

Item 13

13_1a. The experimenter will sugar the cake.

13_1b. The experimenter will sugar the flan.

13_2a. The experimenter has sugared the cake.

13_2b. The experimenter has sugared the flan.

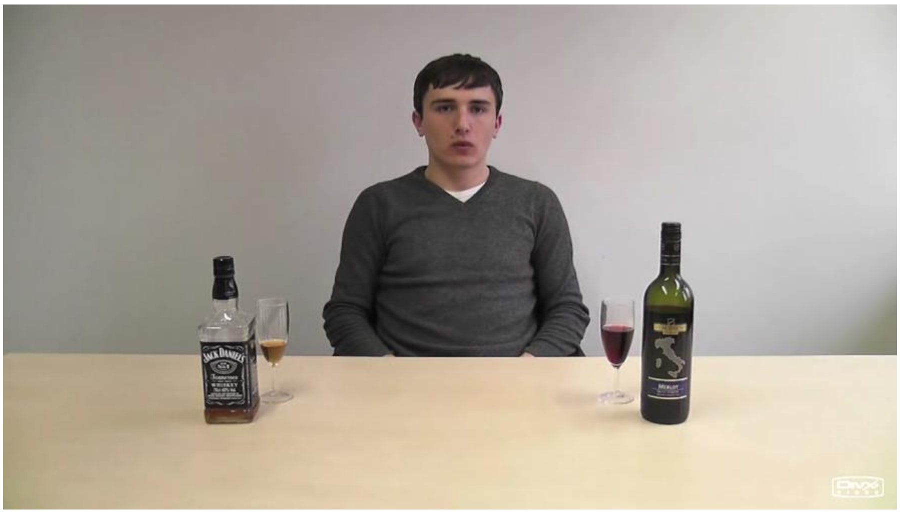

Item 14

14_1a. The experimenter will taste the whisky.

14_1b. The experimenter will taste the red wine.

14_2a. The experimenter has tasted the whisky.

14_2b. The experimenter has tasted the red wine.

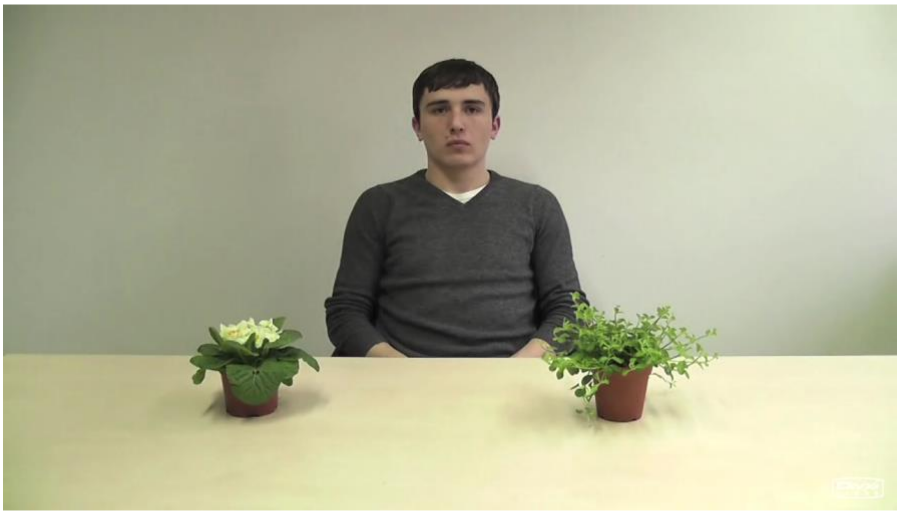

Item 15

15_1a. The experimenter will mist the plant.

15_1b. The experimenter will mist the flower.

15_2a. The experimenter has misted the plant.

15_2b. The experimenter has misted the flower.

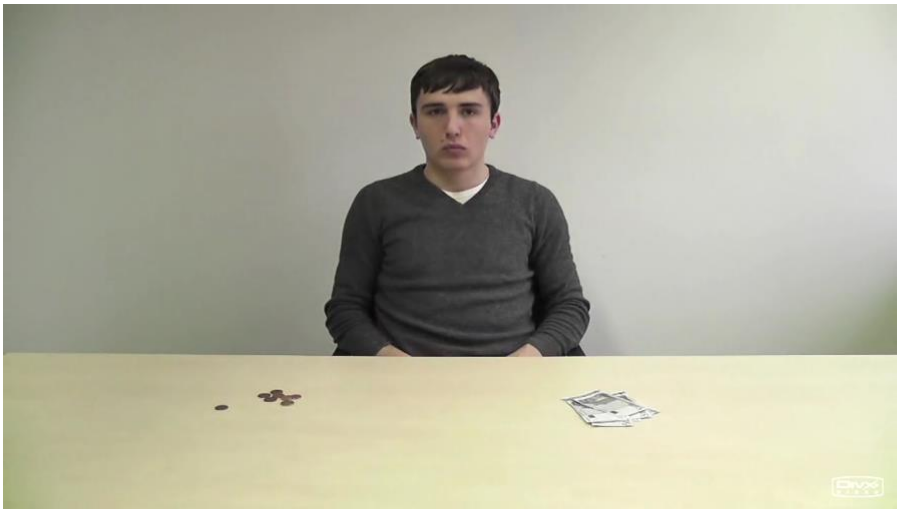

Item 16

16_1a. The experimenter will count the coins.

16_1b. The experimenter will count the bills.

16_2a. The experimenter has counted the coins.

16_2b. The experimenter has counted the bills.

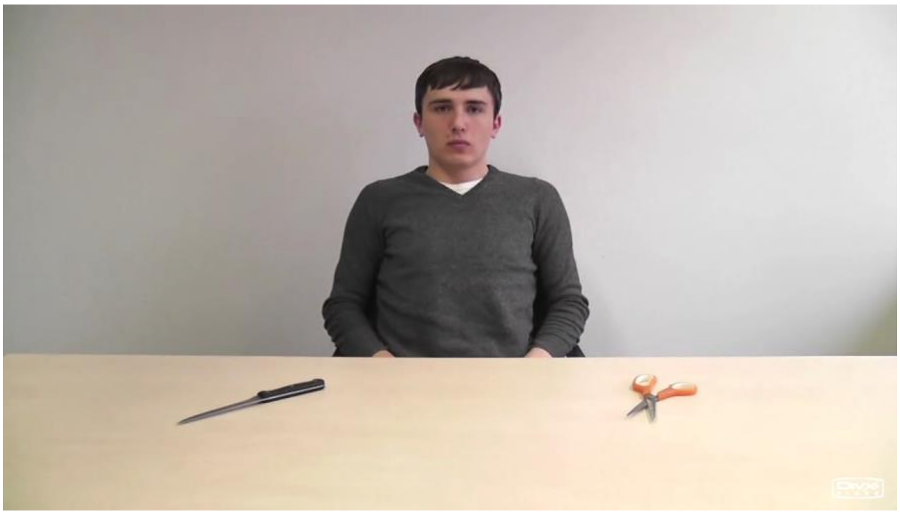

Item 17

17_1a. The experimenter will sharpen the knife.

17_1b. The experimenter will sharpen the scissors.

17_2a. The experimenter has sharpened the knife.

17_2b. The experimenter has sharpened the scissors.

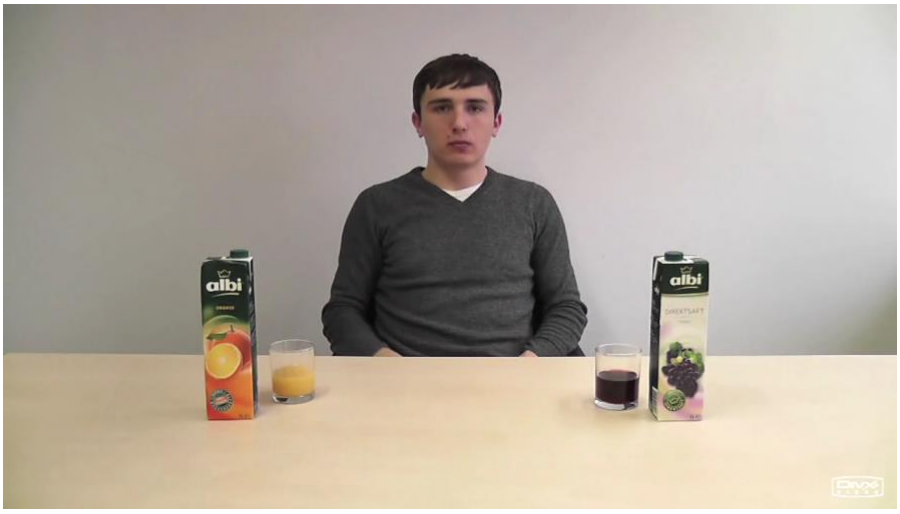

Item 18

18_1a. The experimenter will try the grape juice.

18_1b. The experimenter will try the orange juice.

18_2a. The experimenter has tried the grape juice.

18_2b. The experimenter has tried the orange juice.

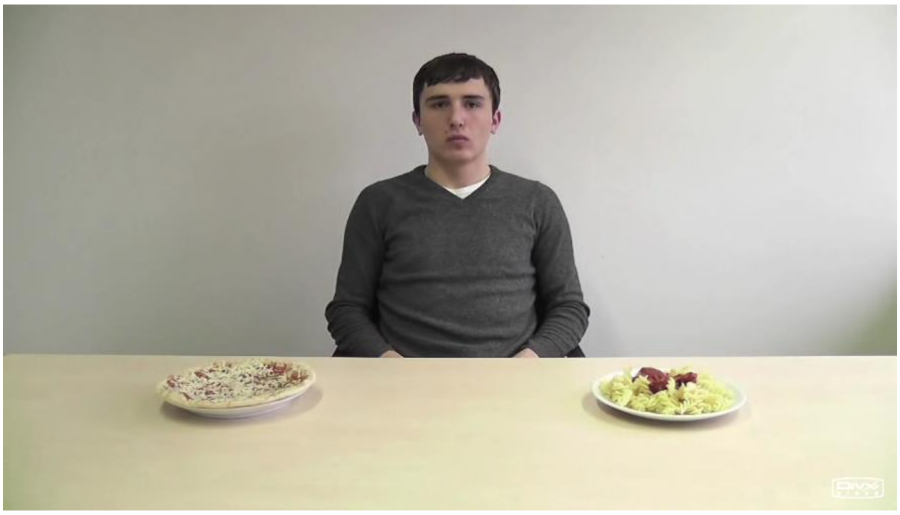

Item 19

19_1a. The experimenter will pepper the pizza.

19_1b. The experimenter will pepper the pasta.

19_2a. The experimenter has peppered the pizza.

19_2b. The experimenter has peppered the pasta.

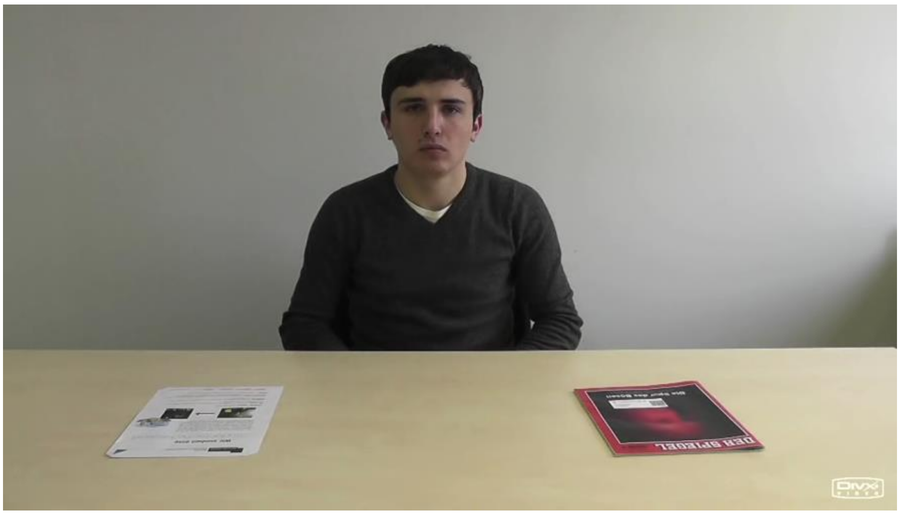

Item 20

20_1a. The experimenter will look at the magazine.

20_1b. The experimenter will look at the advertisement.

20_2a. The experimenter has looked at the magazine.

20_2b. The experimenter has looked at the advertisement.

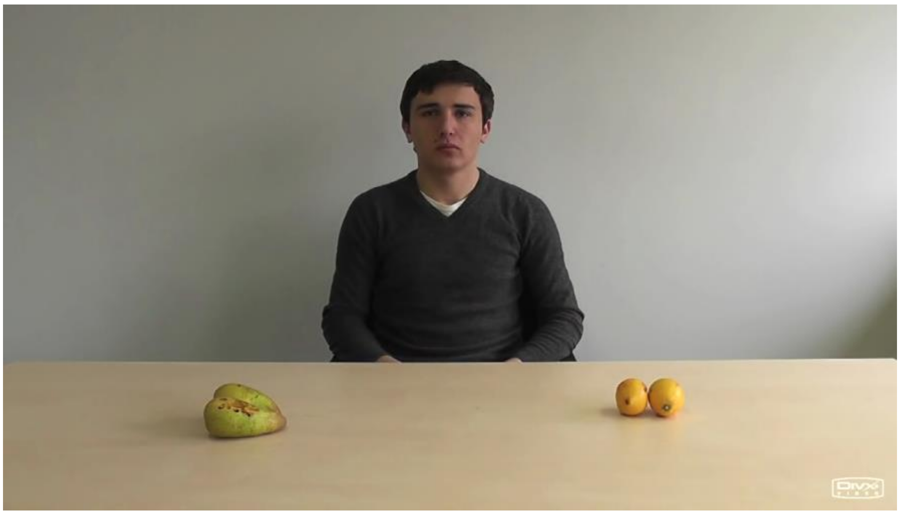

Item 21

21_1a. The experimenter will wipe the lemons.

21_1b. The experimenter will wipe the pears.

21_2a. The experimenter has wiped the lemons.

21_2b. The experimenter has wiped the pears.

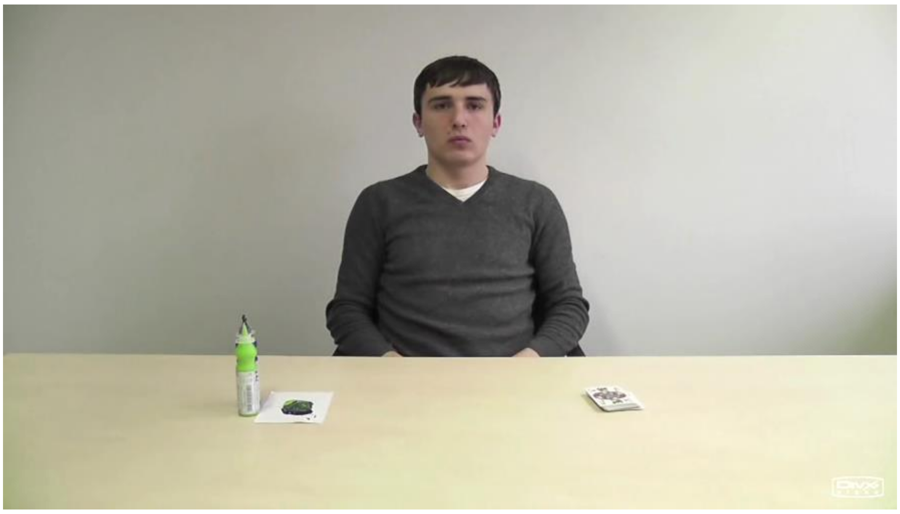

Item 22

22_1a. The experimenter will mix the paint.

22_1b. The experimenter will mix the cards.

22_2a. The experimenter has mixed the paint.

22_2b. The experimenter has mixed the cards.

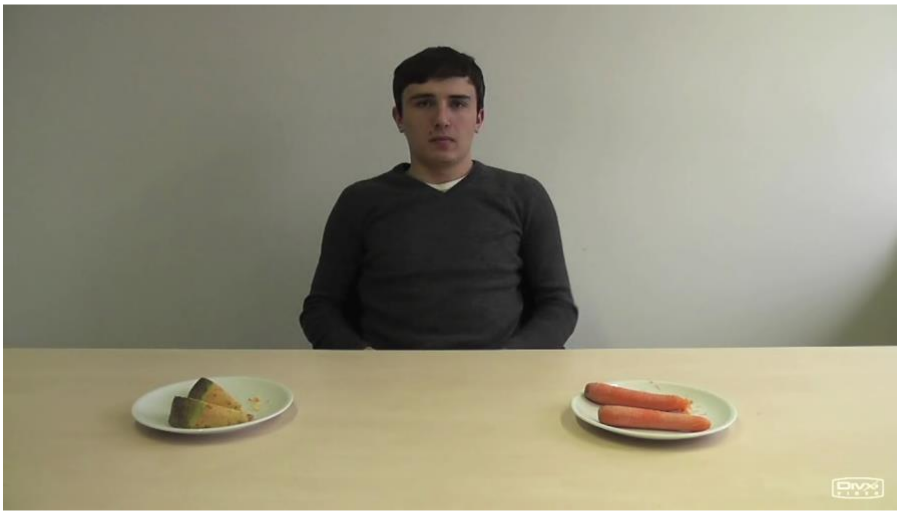

Item 23

23_1a. The experimenter will grate the carrots.

23_1b. The experimenter will grate the turnips.

23_2a. The experimenter has grated the carrots.

23_2b. The experimenter has grated the turnips.

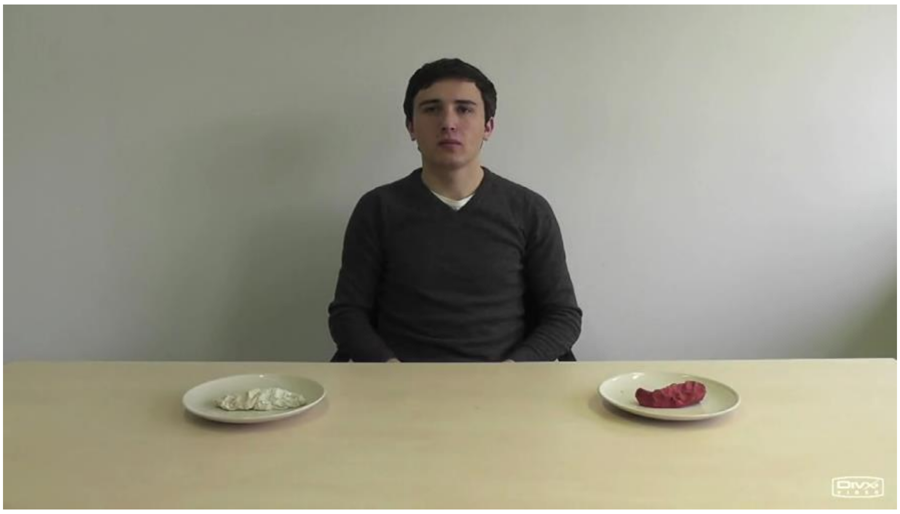

Item 24

24_1a. The experimenter will knead the dough.

24_1b. The experimenter will knead the clay.

24_2a. The experimenter has kneaded the dough.

24_2b. The experimenter has kneaded the clay.
